# Human Colon Mucosal Biofilms and Murine Host Communicate via Altered mRNA and microRNA Expression during Cancer

**DOI:** 10.1128/mSystems.00451-19

**Published:** 2020-01-14

**Authors:** Sarah Tomkovich, Raad Z. Gharaibeh, Christine M. Dejea, Jillian L. Pope, Jinmai Jiang, Kathryn Winglee, Josee Gauthier, Rachel C. Newsome, Ye Yang, Anthony A. Fodor, Thomas D. Schmittgen, Cynthia L. Sears, Christian Jobin

**Affiliations:** a Department of Medicine, University of Florida, Gainesville, Florida, USA; b Department of Infectious Diseases and Immunology, University of Florida, Gainesville, Florida, USA; c Bloomberg-Kimmel Institute of Immunotherapy, Sidney Kimmel Comprehensive Cancer Center, Johns Hopkins Medical Institutions, Baltimore, Maryland, USA; d Department of Oncology and Medicine, Johns Hopkins School of Medicine, Baltimore, Maryland, USA; e Department of Bioinformatics and Genomics, University of North Carolina at Charlotte, Charlotte, North Carolina, USA; f Department of Pharmaceutics, College of Pharmacy, University of Florida, Gainesville, Florida, USA; University of Southampton

**Keywords:** colorectal cancer, transcriptomics, microRNAs, microbiota, biofilm, germfree

## Abstract

Disrupted interactions between host and intestinal bacteria are implicated in colorectal cancer (CRC) development. However, activities derived from these bacteria and their interplay with the host are unclear. Here, we examine this interplay by performing mouse and microbiota RNA sequencing on colon tissues and 16S and small RNA sequencing on stools from germfree (GF) and gnotobiotic *Apc^Min^*^Δ^*^850/+^*;*Il10^−/−^* mice associated with microbes from biofilm-positive human CRC tumor (BF+T) and biofilm-negative healthy (BF-bx) tissues. The bacteria in BF+T mice differentially expressed (DE) >2,900 genes, including genes related to bacterial secretion, virulence, and biofilms but affected only 62 host genes. Small RNA sequencing of stools from these cohorts revealed eight significant DE host microRNAs (miRNAs) based on biofilm status and several miRNAs that correlated with bacterial taxon abundances. Additionally, computational predictions suggest that some miRNAs preferentially target bacterial genes while others primarily target mouse genes. 16S rRNA sequencing of mice that were reassociated with mucosa-associated communities from the initial association revealed a set of 13 bacterial genera associated with cancer that were maintained regardless of whether the reassociation inoculums were initially obtained from murine proximal or distal colon tissues. Our findings suggest that complex interactions within bacterial communities affect host-derived miRNA, bacterial composition, and CRC development.

**IMPORTANCE** Bacteria and bacterial biofilms have been implicated in colorectal cancer (CRC), but it is still unclear what genes these microbial communities express and how they influence the host. MicroRNAs regulate host gene expression and have been explored as potential biomarkers for CRC. An emerging area of research is the ability of microRNAs to impact growth and gene expression of members of the intestinal microbiota. This study examined the bacteria and bacterial transcriptome associated with microbes derived from biofilm-positive human cancers that promoted tumorigenesis in a murine model of CRC. The murine response to different microbial communities (derived from CRC patients or healthy people) was evaluated through RNA and microRNA sequencing. We identified a complex interplay between biofilm-associated bacteria and the host during CRC in mice. These findings may lead to the development of new biomarkers and therapeutics for identifying and treating biofilm-associated CRCs.

## Abstract (precis)

Bacteria and bacterial biofilms have been implicated in colorectal cancer (CRC), but it is still unclear what genes these microbial communities express and how they influence the host. MicroRNAs regulate host gene expression and have been explored as potential biomarkers for CRC. An emerging area of research is the ability of microRNAs to impact growth and gene expression of members of the intestinal microbiota. This study examined the bacteria and bacterial transcriptome associated with microbes derived from biofilm-positive human cancers that promoted tumorigenesis in a murine model of CRC. The murine response to different microbial communities (derived from CRC patients or healthy people) was evaluated through RNA and microRNA sequencing. We identified a complex interplay between biofilm-associated bacteria and the host during CRC in mice. These findings may lead to the development of new biomarkers and therapeutics for identifying and treating biofilm-associated CRCs.

## INTRODUCTION

Numerous 16S rRNA and shotgun metagenomic studies have demonstrated that colorectal cancer (CRC) patients have an altered intestinal microbiota compared to healthy controls (1, 2). Colibactin-producing Escherichia coli, enterotoxigenic Bacteroides fragilis, and Fusobacterium nucleatum among others, are implicated in CRC pathogenesis due to their abilities to produce genotoxins and adhesins which promote proliferation and modulate immune responses in preclinical models (3, 4). How these bacteria interact with the rest of the complex microbiota to influence CRC initiation and/or progression is still unclear. In fact, recent studies with F. nucleatum suggest these bacteria may be associated with later stages of disease and have less of an influence on CRC initiation (5–8). Testing the functional role of human CRC-associated bacterial communities in chemically induced mouse models of CRC have led to mixed results (9, 10). One group demonstrated stool communities from either individual CRC or healthy patients promoted polyp formation depending on the composition of the microbiota that established in mice (9). Another recent report revealed an increased tumorigenic phenotype in mice that received stools pooled from multiple CRC patients compared to stools from controls (10).

The lack of a consensus carcinogenic CRC-associated microbiota from patient stools suggests that other factors, including how the bacteria are organized/located or the genes they express, may be just as important to CRC pathogenesis. Polymicrobial bacterial biofilms, spanning >200 μm of epithelial surface were recently identified in ∼52% of human CRC patients (11) and also found in ∼13% of the healthy patients who were screened (12). We previously showed that human biofilm-forming bacterial communities from either CRC or healthy patients play a functional role in CRC development in multiple preclinical mouse models, emphasizing the contribution of bacterial organization to CRC (13).

CRC is an evolving disease, characterized by a series of molecular and microbial changes (14–16), suggesting a dynamic interplay between the host and intestinal microbiota as the disease progresses. MicroRNAs (miRNAs) have emerged as potential mediators of these host-microbe interactions with their ability to modulate both host (17) and bacterial genes, which can result in shifts in microbiota composition (18, 19). In turn, the microbiota is able to modulate host miRNA expression (18, 20, 21), with F. nucleatum targeting several miRNAs related to CRC pathogenesis (5, 22). However, it is uncertain how human CRC-associated microbial communities as a whole impact fecal miRNA expression and whether host miRNAs affect bacterial composition/gene expression during CRC.

To examine the bacterial activities associated with biofilm-positive microbes from CRC patients, we examined mouse and bacterial gene expression from colon tissues and mouse small RNA sequencing from stools collected from biofilm-positive associated *Apc^Min^*^Δ^*^850/+^*;*Il10^−/−^* mice. We found that a number of bacterial virulence genes were increased in biofilm-positive communities and identified a conserved core group of transmissible biofilm-positive associated bacteria. Additionally, we demonstrate that biofilm status and CRC development alter miRNA expression and specific miRNAs correlate with biofilm-positive associated taxa.

## RESULTS

### Bacterial activities associated with biofilm status.

**FIG 1:**
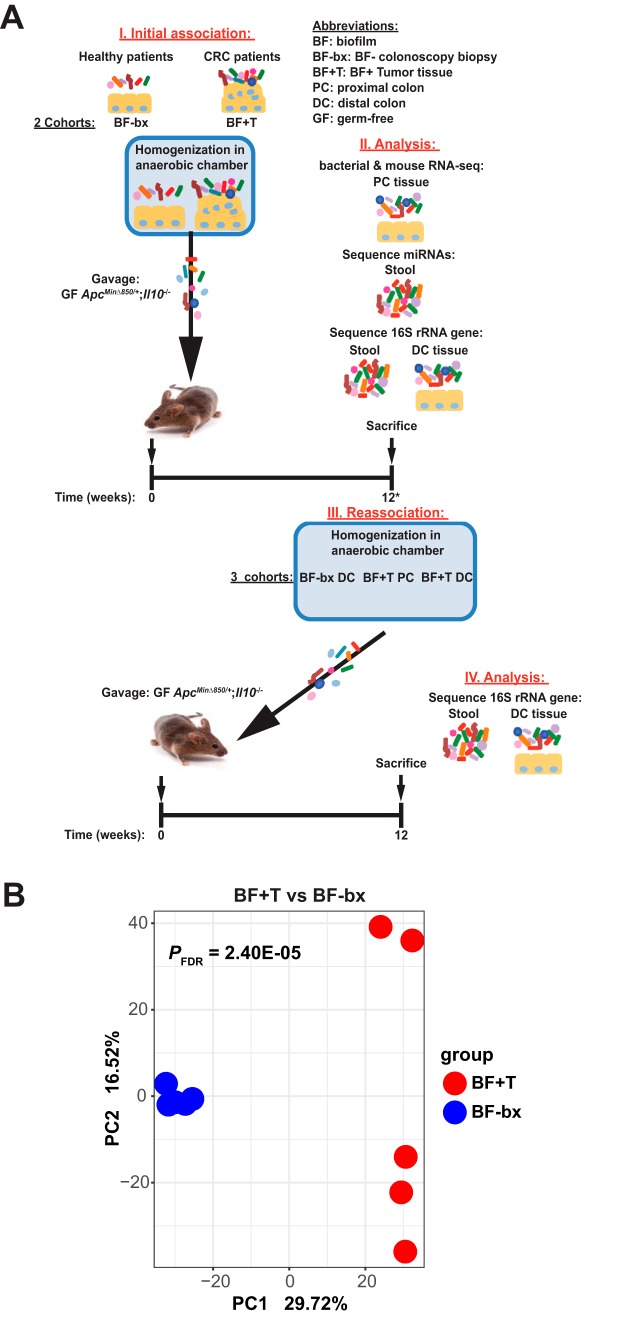
Experimental design and microbial community gene expression associated with biofilm status (related to Materials and Methods). (A) Schematic showing the setup of the gnotobiotic association (I) and reassociation (III) experiments, along with the analyses done on the stool and tissue samples (II and IV) at the end of the 12-week experiments. Twelve-week stool and/or DC tissue samples were used for RNA, miRNA, and 16S rRNA sequencing analyses (II). Tissue was collected from 12-week-associated BF-bx mice and 16- to 20-week-associated BF+T mice to make the reassociation inoculums (III). (B) PCA of bacterial transcriptomes from BF+T- and BF-bx-associated *Apc^Min^*^Δ^*^850/+^*;*Il10^−/−^* mice generated from Trinity *de novo* assembly (*N* = 5 for BF+T and BF-bx).

In order to elucidate microbial activities associated with biofilm-forming bacteria derived from human CRC patients that promote tumorigenesis in *Apc^Min^*^Δ^*^850/+^*;*Il10^−/−^* mice (13), we characterized mouse and microbial gene expression from colon tissue snips using RNA sequencing (see Fig. 1AI and II for experimental design). Principal-component analysis (PCA) of microbial community gene expression detected by both our *de novo* assembly and aligning the microbial transcriptome sequencing (RNA-seq) reads to the human gut microbiome integrated gene catalog (IGC) showed separate clustering of biofilm-positive CRC tumor tissue (BF+T) associated *Apc^Min^*^Δ^*^850/+^*;*Il10^−/−^* mice from biofilm-negative healthy patient tissue (BF-bx) associated mice (Fig. 1B and Fig. S2A in the supplemental material, respectively). Bacterial metatranscriptomic analysis found 2,918 significant differentially expressed (DE) genes (false-discovery rate-adjusted *P* value [*P*_FDR_] < 0.05), the majority of which were increased in BF+T mice (2,739 increased genes and 179 decreased genes [see Table S1 at https://figshare.com/s/bd06b560c635de3ac830]) compared to BF-bx mice. Pathways related to protein export, bacterial secretion systems, carbon fixation, flagellar assembly, and biosynthesis of amino acids were increased in BF+T mice compared to BF-bx mice (Table 1 and Fig. S2B and C). Additional genes related to virulence and biofilm formation, including stress response, toxins, iron acquisition, mucin cleavage/transport, outer membrane polysaccharide importers, and adhesins were also significantly increased in BF+T mice (see Table S1 at https://figshare.com/s/bd06b560c635de3ac830). Increased toxin genes included Clostridium difficile toxins A and B, Clostridium perfringens Mu toxin, and E. coli colibactin (*clbG* and *clbI*). Weighted gene coexpression network analysis (23) identified 34 hub genes (see Table S1 at https://figshare.com/s/bd06b560c635de3ac830) from modules detected in BF+T mice and included outer membrane proteins involved in protein export and heat shock proteins involved in the stress response.

**TABLE 1:** Pathways enriched in BF+T samples using KEGG pathways from Trinity assembly or HUMAnN analysis of RNA-seq reads

Method and pathway	*P* value	*P*FDR
Trinity assembly		
Carbon fixation pathways in prokaryotes	1.06e−05	0.0006
Protein export	0.0002	0.0051
Bacterial secretion system	0.0002	0.0051
Valine, leucine, and isoleucine biosynthesis	0.0004	0.007
2-Oxocarboxylic acid metabolism	0.001	0.0131
		
HUMAnN analysis		
2-Oxocarboxylic acid metabolism	1.41e−05	0.0007
Inositol phosphate metabolism	1.42e−05	0.0007
Biosynthesis of amino acids	0.0004	0.0163
Flagellar assembly	0.001	0.0204
Protein export	0.0021	0.035

**FIG 2:**
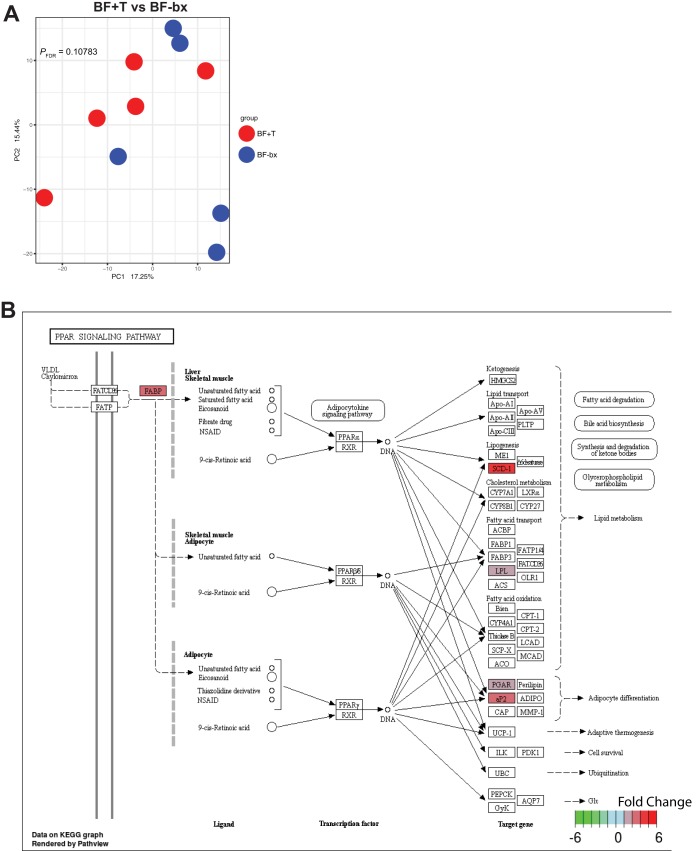
Mouse gene expression affected by biofilm status. (A) PCA of mouse transcriptomes from BF+T and BF-bx mice (*N* = 5 per group). (B) PPAR signaling pathway depicting genes significantly increased in BF+T associated mice in red. See Table S2 at https://figshare.com/s/4b593b780f756a4acf69 for a list of all significant DE genes in BF+T versus BF-bx associated mice. Boxes without color denote no significant change.

Despite the high number of microbial genes with increased expression in BF+T microbial communities, no separation by PCA analysis was found at the host gene expression level, and only 62 significant DE genes (Fig. 2A; see also Table S2 at https://figshare.com/s/4b593b780f756a4acf69) between BF+T and BF-bx mice were detected. Instead, the host was more responsive to the microbiota in general as opposed to the type of microbiota, since the host transcriptomes of either BF+T- or BF-bx-associated mice clustered separately from germfree (GF) mice (Fig. S1A and B). There were >3,000 significant DE host genes (∼2,000 upregulated, ∼1,300 downregulated) in the BF+T and BF-bx groups compared to GF mice (see Tables S3 at https://figshare.com/s/163676e591c87b0d6c35, S4 at https://figshare.com/s/652055bdb15e48b866ef, and S5 at https://figshare.com/s/c9adfcd56af278666c55) and pathway analysis revealed that the majority of upregulated genes in colonized mice belonged to immune-related pathways (Fig. S1C and D). Only the peroxisome proliferator-activated receptor (PPAR) signaling pathway was significantly upregulated (*P* = 1.88e−05, *P*_FDR_ = 0.004) in BF+T mice compared to BF-bx mice. The majority of the significant DE genes were upregulated (56 versus 6 downregulated) in BF+T mice and included genes related to lipid metabolism, iron scavenging, and the extracellular matrix (see Table S2 at https://figshare.com/s/4b593b780f756a4acf69). Cross-referencing the list of upregulated genes with The Cancer Genome Atlas (TCGA) colorectal microarray data using Oncomine (24) revealed 10 of the upregulated genes were also significantly overexpressed in colon adenocarcinomas compared to control tissues with SCD (stearoyl-coenzyme A [CoA] desaturase, PPAR pathway member), MMP10, and SLC22A3 increased more than 2-fold (see Table S6 at https://figshare.com/s/7248a5727e5972879086).

**FIG 3:**
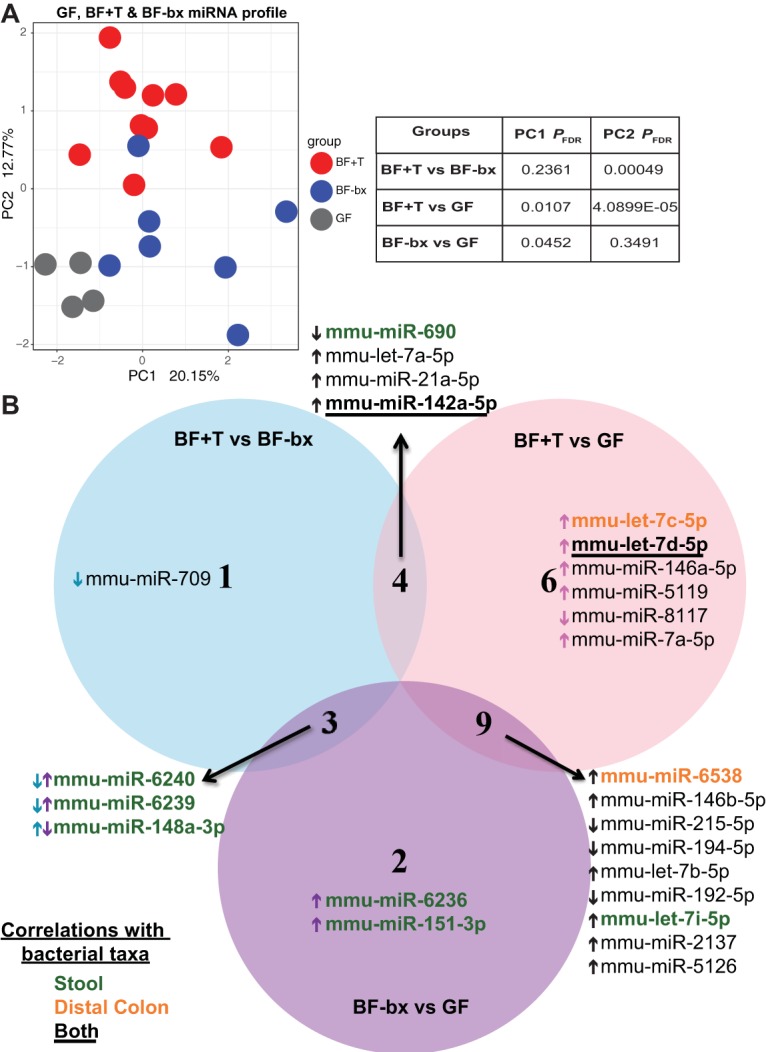
Fecal miRNA profiles and significant DE miRNAs according to biofilm status. (A) PCA plot of log-normalized mature miRNA counts in GF, BF-bx, and BF+T *Apc^MinΔ850/+^*;*Il10^−/−^* stool (*N* = 4, 7, and 10 for GF, BF-bx, and BF+T, respectively). Symbols represent miRNAs from individual mice for BF-bx and BF+T groups. For the GF group, symbols represent miRNAs pooled from 2 to 5 mice (13 mice total, see Materials and Methods). (B) Significant (*P*_FDR_ < 0.05) DE miRNA between the three experimental groups. BF+T microbiota elicited more host miRNAs than the GF and BF-bx microbiota with six miRNAs uniquely DE in response to it. The arrows next to miRNA names indicate whether the miRNA is increased (↑) or decreased (↓) in the first group listed relative to the second for each comparison. Blue arrows indicate direction of miRNA expression in BF+T mice compared to BF-bx mice. Pink arrows indicate direction of miRNA expression in BF+T mice compared to GF mice. Purple arrows indicate direction of miRNA expression in BF-bx mice compared to GF mice. Black arrows indicate direction of miRNAs that are shared between comparison groups and go in the same direction. The miRNAs that are significantly correlated with taxa identified from 16S rRNA sequencing of the stool are highlighted in green, those that significantly correlated with taxa identified from the DC tissue are highlighted in orange, and those correlating with both are in black font and underlined (total of 11 miRNAs).

10.1128/mSystems.00451-19.1 FIG S1 Mouse genes upregulated in human tissue microbiota-associated *Apc^Min^*^Δ^*^850^*;*Il10^−/−^* mice. This figure is related to Fig. 3. (A) PCA of mouse transcriptomes from BF+T-associated and GF mice (*N* = 5 and 3, respectively). (B) PCA of mouse transcriptomes from BF-bx-associated and GF mice (*N* = 5 and 3, respectively). (C and D) Word cloud analysis of KEGG pathways upregulated in BF+T-associated mice (C) or BF-bx-associated mice (D) compared to GF mice. The pathways with *P*_FDR_ of <0.05 are depicted in red font, and the font size corresponds to the –log FDR. See Tables S3 at https://figshare.com/s/163676e591c87b0d6c35 and S4 at https://figshare.com/s/652055bdb15e48b866ef for lists of all significant DE genes in BF+T- and BF-bx-associated mice compared to GF mice, respectively. Download FIG S1, PDF file, 1.6 MB. Copyright © 2020 Tomkovich et al. 2020 Tomkovich et al. This content is distributed under the terms of the Creative Commons Attribution 4.0 International license.

10.1128/mSystems.00451-19.2 FIG S2 (A) PCA of bacterial transcriptomes from BF+T- and BF-bx-associated *Apc^Min^*^Δ^*^850/+^*;*Il10^−/−^* mice generated from aligning the reads to the human gut microbiome integrated gene catalog (IGC) show similar clustering to that obtained from *de novo* assembly (*N* = 5 for BF+T and BF-bx). Bacterial pathways increased in BF+T mice. (B and C) Visualization of significant DE genes within the bacterial secretion system (B) and flagellar assembly (C). KEGG pathways created with Pathview. Genes that were increased in BF+T mice are depicted in red, while decreased genes are depicted in green. See Table S1 at https://figshare.com/s/bd06b560c635de3ac830 for a list of all significant DE genes. Download FIG S2, PDF file, 0.4 MB. Copyright © 2020 Tomkovich et al. 2020 Tomkovich et al. This content is distributed under the terms of the Creative Commons Attribution 4.0 International license.

### Biofilm status alters the fecal miRNA profile.

Given the differential bacterial and host gene expression observed in BF+T mice that developed colon tumors and the potential of miRNAs to modulate interkingdom interactions (18), we next profiled stool miRNA expression. To examine the interplay between miRNAs and bacterial communities derived from human CRC or healthy patients, we sequenced the fecal small RNAs from GF, BF-bx, and BF+T *Apc^Min^*^Δ^*^850/+^*;*Il10^−/−^* mice. The PCA plot shows clear separation between GF mice and BF-bx or BF+T-associated mice, demonstrating that microbial colonization alters the fecal miRNA profile in *Apc^Min^*^Δ^*^850/+^*;*Il10^−/−^* mice (Fig. 3A; see also Table S7 at https://figshare.com/s/b7d7ff960f787eb31954). Biofilm/cancer status of the initial human-derived microbes also modulates miRNA expression since PCA analysis demonstrates separation of BF+T and BF-bx miRNAs (Fig. 3A; see also Table S7 at https://figshare.com/s/b7d7ff960f787eb31954). Pairwise comparisons between the three groups of mice revealed that 25 unique miRNAs were significantly DE (out of 142 total detected) (Fig. 3B; see also Tables S7 at https://figshare.com/s/b7d7ff960f787eb31954, S8 at https://figshare.com/s/2eaad72eea8a5890f7f2, and S9 at https://figshare.com/s/20a8d9c108e990242bc7). Next, we compared the significantly different miRNAs in BF+T or BF-bx versus GF mice and found that nine significant DE miRNAs overlapped (mmu-miR-6538, -146b-5p, -215-5p, -194-5p, -192-5p, -2137, and -5126 and mmu-let-7b-5p and mmu-let-7i-5p), suggesting host miRNAs targeting the microbiota (Fig. 3B; see also Tables S8 at https://figshare.com/s/2eaad72eea8a5890f7f2 and S9 at https://figshare.com/s/20a8d9c108e990242bc7). We were also able to identify eight miRNAs (mmu-miR-709, -690, -21a-5p, -142a-5p, -6240, -6239, and -148a-3p and mmu-let-7a-5p) that were significantly DE according to biofilm status (BF+T versus BF-bx [Fig. 3B; see also Table S7 at https://figshare.com/s/b7d7ff960f787eb31954]). These findings suggest that the microbiota modulates host miRNA expression. Together, both microbiota composition and disease status drive miRNA expression (BF+T versus BF-bx DE miRNAs).

### Fecal miRNAs correlate with specific bacterial taxa and target bacterial and mouse genes.

**FIG 4:**
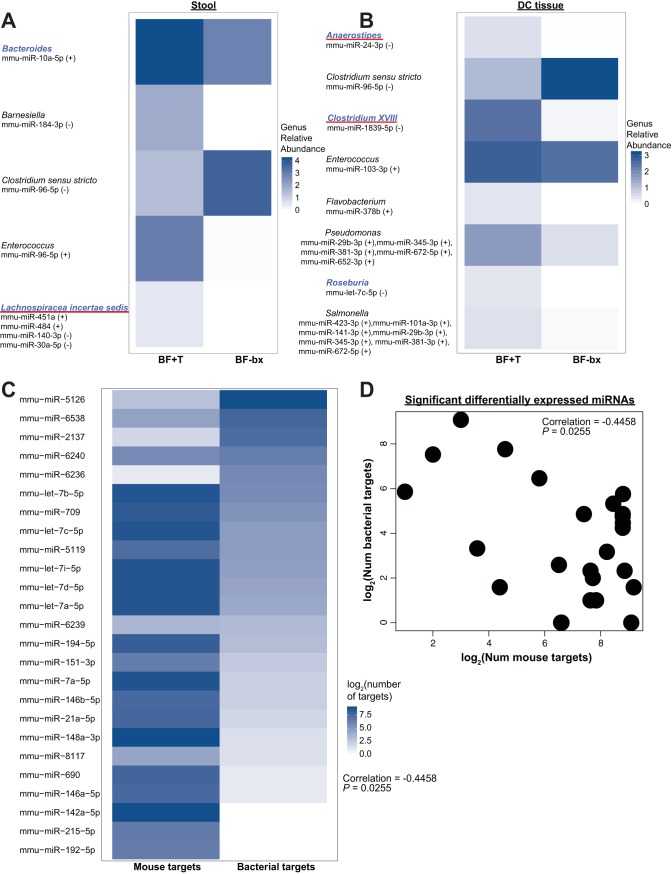
Microbial relative abundance at the genus level correlates with specific miRNAs. (A and B) Heatmap depicting the mean log_10_-normalized relative abundance of genera within the stool (A) or distal colon tissue (B) compartments that have significant (*P*_FDR_ < 0.05) correlation with miRNA expression. The name of each miRNA is shown below the genus it correlates with. Genera in blue font are significantly different between BF-bx and BF+T *Apc^MinΔ850/+^*;*Il10^−/−^* mice. The red underlined genera were significantly different based on biofilm status in both the initial association and reassociation experiments. The direction of correlation is shown within parentheses. Relative abundance data are from the subset of BF-bx and BF+T mice that were used for miRNA sequencing (*n* = 7 for BF-bx; *n* = 10 for BF+T). See Tables S10 at https://figshare.com/s/55e156ec52d9d3e53394 and S11 at https://figshare.com/s/1c47b438d806062915c9 for the full list of miRNAs that correlate with bacterial taxa and the corresponding correlation coefficients. (C) Heatmap comparing the log_2_-transformed number of predicted bacterial versus mouse gene targets for the miRNAs that were significantly DE between the GF, BF-bx, or BF+T group. There is a significant negative correlation (Pearson) between the number of predicted bacterial versus mouse gene targets for the set of significant DE miRNAs. (D) Scatter plot demonstrating the significant negative Pearson correlation between the log_2_-transformed number of mouse versus bacterial gene targets where each circle represents a unique mouse miRNA that was significantly DE between GF, BF-bx, or BF+T group.

We next examined whether the fecal miRNAs of human microbiota-associated mice correlated with bacterial taxa previously identified in the mice (13). We identified 11 miRNAs that were significantly DE between GF, BF-bx, and BF+T mice and correlated with the relative abundances of bacterial taxa in the stool, distal colon tissue, or both compartments (Fig. 3B [colored green, orange, or underlined]; see also Tables S10 at https://figshare.com/s/55e156ec52d9d3e53394 and S11 at https://figshare.com/s/1c47b438d806062915c9). Additionally, we identified miRNAs that although not significantly DE between groups, correlated with five and eight bacterial genera in the stool and distal colon tissues, respectively (Fig. 4A and B; see also Tables S10 at https://figshare.com/s/55e156ec52d9d3e53394 and S11 at https://figshare.com/s/1c47b438d806062915c9). Five of these genera (*Bacteroides*, *Lachnospiracea incertae sedis*, *Anaerostipes*, *Clostridium* XVIII, and *Roseburia*) were significantly increased in BF+T mice (Fig. 4A and B; see also Tables S10 at https://figshare.com/s/55e156ec52d9d3e53394 and S11 at https://figshare.com/s/1c47b438d806062915c9). Furthermore, mmu-miR-140-3p correlated with *Lachnospiraceae incertae sedis* abundance and tumor number, both of which were increased in BF+T mice (Fig. 4A and Fig. S3A). Thus, CRC-associated microbial communities elicit a specific host miRNA profile and maintain a subset of miRNAs that correlate with specific microbial taxa.

10.1128/mSystems.00451-19.3 FIG S3 miRNA expression correlation with tumor numbers and miRNA correlation with mouse and bacterial gene targets are not due to chance. This figure is related to Fig. 4. (A) Spearman’s rank correlation with *P*_FDR_ is shown. Three BF-bx samples are at the 0,0 coordinates and are represented as one circle; all other circles represent individual mice. *N* = 5 and 10 for BF-bx and BF+T, respectively. Two BF-bx samples were excluded due to colon collection method (see Materials and Methods). Mmu-miR-140-3p also correlates with *Lachnospiraceae incertae sedis* abundance in the stool compartment (see Fig. 4 and Table S10 at https://figshare.com/s/55e156ec52d9d3e53394). (B and C) Correlating the number of predicted bacterial versus mouse gene targets for 100 randomly selected sets of 25 mouse miRNAs from the list of 127 miRNAs that were detected in at least 25% of the samples (excluding those shown in Fig. 4C) show no significant correlations. (B) Boxplot showing the correlation coefficients and (C) boxplot showing −log_10_ *P* values for the correlation in panel B. The red dotted line marks *P* = 0.05, values above the line indicate *P* < 0.05, and values below the line indicate *P* > 0.05. Download FIG S3, PDF file, 0.2 MB. Copyright © 2020 Tomkovich et al. 2020 Tomkovich et al. This content is distributed under the terms of the Creative Commons Attribution 4.0 International license.

Interestingly, computationally predicting the bacterial genes targeted by the significant miRNAs revealed several miRNAs (mmu-miR-2137, mmu-miR-5126, and mmu-miR-6538) that primarily target bacterial genes (see Table S12 at https://figshare.com/s/33fb65da16763a5e3347). In contrast, other miRNAs (mmu-let-7s, mmu-miR-21a-5p, mmu-miR-142a-5p, mmu-miR-148a-3p, mmu-miR-194-5p, mmu-miR-690, and mmu-miR-709) are predicted to primarily target mouse genes (see Table S13 at https://figshare.com/s/be3f38607b2e9875e7f6), and there is a significant negative correlation between the number of predicted bacterial versus mouse gene targets for the significant miRNAs we identified (Fig. 4C and D and Fig. S3B and C). Taken together, these predictions suggest that specific miRNAs have differential roles in mediating host-microbe interactions, with some mainly modulating host gene expression and others primarily impacting bacterial gene expression and/or abundance.

### A core BF+T microbiota is transmissible.

We previously showed that carcinogenic properties are retained over time as microbial inoculums from homogenized mouse colon tissues collected from the initial BF+T but not BF-bx tissue-associated microbes promoted tumors in new cohorts of GF *Apc^Min^*^Δ^*^850/+^*;*Il10^−/−^* mice (13) (Fig. 1A.III).

**FIG 5:**
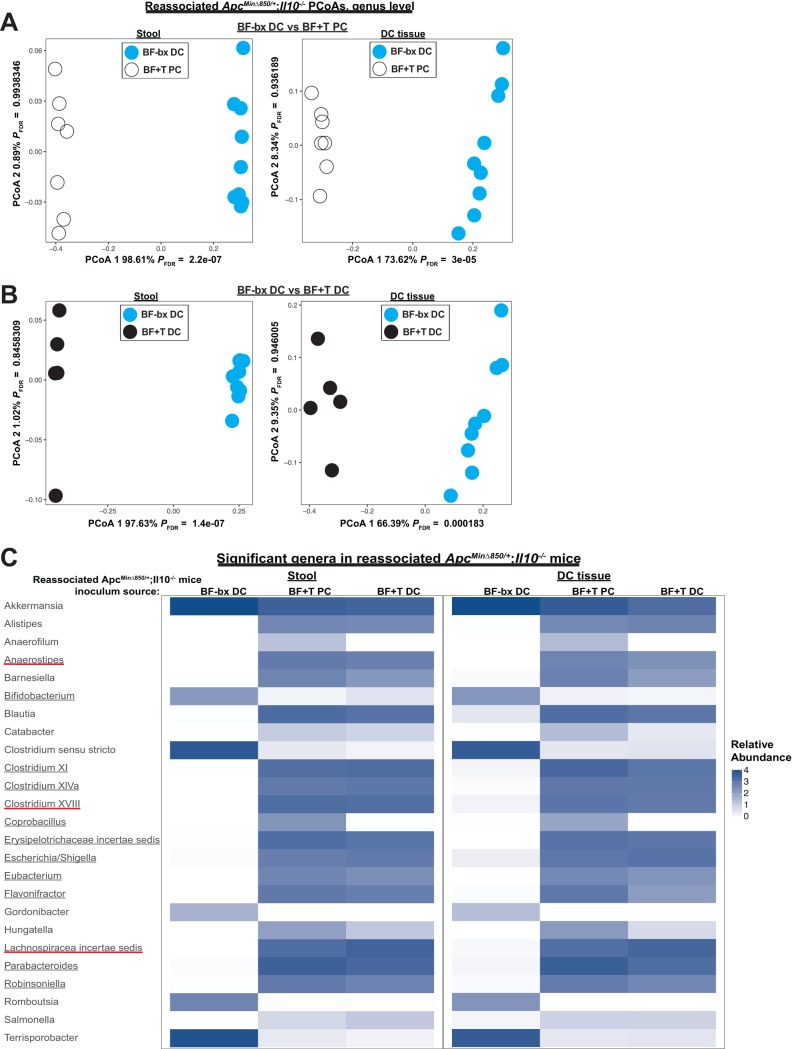
Biofilm status is associated with microbiota changes in reassociated *Apc^MinΔ850/+^*;*Il10^−/−^* mice. (A and B) PCoAs of BF-bx and BF+T reassociated mice, with the composition of the stool and DC tissue compartment on the left and right, respectively (*N* = 9, 7, and 5 for BF-bx DC, BF+T PC, and BF+T DC, respectively). (A) BF+T PC reassociation compared to the BF-bx DC reassociation. (B) BF+T DC reassociation compared to the BF-bx DC reassociation. (C) Heatmap depicting the mean log_10_-normalized relative abundances of genera that were significantly different in the stool and/or DC tissues of reassociated mice inoculated with murine colon tissue homogenates derived from human BF+T or BF-bx tissue-associated mice. The underlined genera were significantly different based on biofilm status in both the initial association and the reassociation, and the red underlines represent the three genera that correlated with specific miRNAs (Fig. 4A and B).

We next examined microbial compositional differences between BF+T and BF-bx reassociated mice. 16S rRNA sequencing revealed separation of stool and distal colon (DC) tissue microbiota from reassociated mice according to the biofilm status of the initial association (Fig. 5A and B). Thirteen genera were significantly different (12 genera enriched and one genus depleted) in the stool and/or DC tissue of both the BF+T-associated and reassociated *Apc^Min^*^Δ^*^850/+^*;*Il10^−/−^* mice (Fig. 5C), three of which also correlated with specific miRNAs (Fig. 4A and B). Six of these genera (*Clostridium* XVIII, *Erysipelotrichaceae incertae sedis*, *Escherichia/Shigella*, *Eubacterium*, *Parabacteroides*, and *Robinsoniella*) were increased in both the stool and DC tissue of BF+T-associated and reassociated mice compared to BF-bx mice. To determine how much the microbiota composition shifted after reassociation, we compared the microbiotas from the reassociated mice to the initial associated mice, whose tissues were used to generate the inoculums (Fig. S4 and S5). The BF-bx communities were highly transmissible, with no significant differences based on principal-coordinate analysis (PCoA) of the stool and DC tissue communities (Fig. S4). The BF+T communities shifted more after reassociation, with distinct clustering seen in the PCoA (Fig. S5A and B). Depending on the colon region the BF+T inoculum was derived from, there were 5 and 13 significantly different genera within the DC tissue or stool compartment; however, only 1 and 5 of these genera were significantly different based on biofilm status in the initial associations (Fig. S5C and D). We also examined how the location of the colon tissues used to make the BF+T reassociation inoculums impacts community composition by comparing the stool and DC tissue communities from mice reassociated with PC or DC tissue inoculums (Fig. S6A). We found that only 2 and 5 genera were significantly different (Fig. S6B) and only 2 of these genera (*Coprobacillus* and *Holdemania*) differed according to biofilm status in the initial associations. Out of the 12 genera that were increased in BF+T-associated and reassociated mice, only 1 (*Coprobacillus*) was not maintained in both groups of BF+T reassociated mice. Thus, regardless of the murine colon region of origin (proximal or distal), the majority of BF+T microbes (11 genera total; *Anaerostipes*, *Clostridium* XI, *Clostridium* XIVa, *Clostridium* XVIII, *Erysipelotrichaceae incertae sedis*, *Escherichia/Shigella*, *Eubacterium*, *Flavonifractor*, *Lachnospiraceae incertae sedis*, *Parabacteroides*, and *Robinsoniella*) are able to reestablish and promote cancer when transmitted to a new cohort of GF mice. Taken together, the data suggest there is a core set of bacteria and bacterial gene expression associated with biofilm-positive cancers.

10.1128/mSystems.00451-19.4 FIG S4 Bacterial composition of biofilm-negative reassociated mice resembles the initial biofilm-negative association. This figure is related to Fig. 5. (A and B) PCoAs comparing stool (A) and DC tissue (B) bacterial composition between the BF-bx mice and BF-bx DC reassociated mice. (C) Operational taxonomic unit (OTU) level PCoA of the BF-bx mice and BF-bx DC reassociated mice stools generated from rarefied QIIME closed-reference OTUs using unweighted UniFrac distance metric. (D) Suboperational taxonomic unit (sOTU) level PCoA of the BF-bx mice and BF-bx DC reassociated mice stools generated from rarefied Deblur sOTUs using unweighted UniFrac distance metric. For stool bacterial composition (panel A), *N* = 7 and 9 for BF-bx and BF-bx DC, respectively. For DC tissue bacterial composition (panels B to D), *N* = 6 and 9 for BF-bx and BF-bx DC, respectively. The BF-bx DC reassociation inoculum was prepared from additional DC tissue pieces taken from four of the BF-bx-associated mice. Download FIG S4, PDF file, 0.2 MB. Copyright © 2020 Tomkovich et al. 2020 Tomkovich et al. This content is distributed under the terms of the Creative Commons Attribution 4.0 International license.

10.1128/mSystems.00451-19.5 FIG S5 Bacterial composition of biofilm-positive reassociated mice shifts compared to the initial biofilm-positive association, but the majority of candidate biofilm-associated taxa were unaffected. This figure is related to Fig. 5 and Fig. S6. (A and B) PCoAs comparing stool (A) and DC tissue (B) bacterial composition between the BF+T mice and either the BF+T PC (left panel) or BF+T DC (right panel) reassociated mice (*N* = 7, 7, and 5 for BF+T, BF+T PC, and B + T DC, respectively). The BF+T reassociation inoculums were made from either additional PC or DC tissue pieces from four of the BF+T associated mice. (C and D) Heatmaps representing the mean log_10_-normalized relative abundances of genera that were significantly different between the initial association and the reassociation in either the stool (C) or DC tissue (D) compartments. The underlined genera were significantly different in the reassociated mice based on biofilm status in the initial association. Download FIG S5, PDF file, 0.2 MB. Copyright © 2020 Tomkovich et al. 2020 Tomkovich et al. This content is distributed under the terms of the Creative Commons Attribution 4.0 International license.

10.1128/mSystems.00451-19.6 FIG S6 Biofilm-positive reassociated mice establish similar bacterial communities regardless of the colon region used to make the reassociation inoculums. This figure is related to Fig. 5 and Fig. S5. (A) PCoAs comparing the stool and DC tissue bacterial communities from BF+T reassociated mice given an inoculum made from either proximal (BF+T PC) or distal (BF+T DC) colon tissues (*N* = 7 and 5 for BF+T PC and BF+T DC, respectively). (B) Heatmaps depicting the mean log_10_-normalized relative abundances of genera that were significantly different between the two BF+T reassociation groups. The underlined genera were part of the 24 genera significantly different based on biofilm status in the initial association (13), but were not maintained in one of the BF+T reassociation groups. Download FIG S6, PDF file, 0.2 MB. Copyright © 2020 Tomkovich et al. 2020 Tomkovich et al. This content is distributed under the terms of the Creative Commons Attribution 4.0 International license.

## DISCUSSION

In contrast to previous studies that tested the carcinogenicity of CRC-associated microbiotas by gavaging mice with stools from either CRC or healthy control patients (9, 10), mucosa-associated BF+T bacteria retain their carcinogenicity when transplanted into a new set of GF mice, regardless of whether they were extracted from the proximal or distal colon (13). The number of taxa that overlap between the initial association and the reassociation experiments, shown herein (Fig. 5), suggest that there is a core, transmissible, cancer-promoting microbiota associated with biofilm-positive cancers.

Four of the BF+T core transmissible cancer-promoting genera overlap with human stool-derived taxa that established and were associated with cancer in mice. *Parabacteroides* correlated with high tumor numbers in a gnotobiotic azoxymethane (AOM)/dextran sulfate sodium (DSS) C57BL/6 model (9), and *Erysipelotrichaceae*, *Lachnospiraceae*, and a *Clostridium* XIVa derived from CRC patients were associated with polyp formation in an antibiotic-treated AOM C57BL/6 model (10). Additionally, increased *Erysipelotrichaceae*, E. coli, *Lachnospiraceae*, and *Parabacteroides* and decreased *Bifidobacterium* have previously been associated with human CRC patient samples by 16S rRNA gene sequencing studies (1, 25).

Metagenomic predictions generated from 16S rRNA data identified bacterial secretion systems and motility genes associated with human CRC stool communities (26) and host glycan utilization genes correlated with tumor numbers in human stool-associated AOM/DSS mice (9). Recent meta-analysis studies of metagenomes from CRC patient fecal samples identified gluconeogenesis, mucin degradation, and colibactin as associated with the CRC microbiome (27, 28). These bacterial pathways and genes were also increased in our BF+T metatranscriptome. PICRUSt analysis of biofilm-positive versus biofilm-negative human CRC tissues also demonstrated an increased sporulation capacity associated with biofilm-positive CRCs contributed by several taxa, among them the *Lachnospiraceae* family (11). Similarly, herein, we saw genera from the *Lachnospiraceae* family (*Anaerostipes*, *Clostridium* XIVa, *Lachnospiracea incertae sedis*, and *Robinsoniella*) and upregulation of multiple sporulation genes in mice transplanted with the BF+T community. There is also overlap between BF+T community gene expression and human periodontitis polymicrobial metatranscriptomes, particularly for genes related to iron acquisition, flagellar synthesis, and the stress response (29). These findings are interesting since biofilms have also been associated with periodontal disease and F. nucleatum and *Porphyromonas* spp. are associated with both oral biofilms and colon cancer (4, 11, 25, 30).

Multiple genes related to nutrient, envelope, DNA damage, and environmental stress responses were increased in the BF+T community that could be indicative of host immune pressures, but could also be associated with a competitive polymicrobial environment, a feature of biofilms (31). Host iron metabolism changes during inflammation and cancer can promote competition for iron within the intestinal microbiota (32). Multiple iron acquisition genes, including siderophores and transport receptors, were increased in BF+T mice (32). The expression of these metabolic and iron acquisition genes could be indicative of a low-nutrient environment, fostering interbacterial competition.

Bacterial adhesion genes are a critical colonization determinant and may also contribute to biofilm formation, in which attachment to host cells represents a key initiating step (33). There are a number of adhesins expressed in the BF+T community, including type I and IV pili, capsule genes, and proteins that bind to host extracellular matrix (ECM) components such as fibronectin and laminin. On the host side, BF+T mice exhibit upregulation of a laminin subunit and the ECM-degrading matrix metalloproteinase MMP10 (34), suggesting alterations to the host ECM. BF+T communities also expressed numerous moonlighting adhesins (such as flagellin, GroEL, DnaK, and elongation factor Tu), putative multifunctional proteins which have been demonstrated in some bacterial strains to bind host cells, mucin, or ECM components (35). Bacterial adherence has also been identified as an important feature of CRC-associated bacteria, including F. nucleatum (36) and Streptococcus gallolyticus subsp. *gallolyticus* (37), the latter of which is capable of forming biofilms on collagen IV, an ECM component (38). Furthermore, bacteria expressing adhesins that bind to ECM and host glycoproteins may have an additional colonization advantage, as host glycosylation is disrupted during inflammation and cancer with increases in sialylation and fucosylation that can result in decreased host cell adhesion to ECM components (39). Additionally, some of the effects of CRC-associated bacteria may be contact dependent; for example, colibactin-induced DNA damage requires direct contact between the bacteria and epithelial cells (40).

Coupled with its role in facilitating colonization and attachment, mucin also represents a source of nutrition for intestinal bacteria (41). Antibiotic treatment was shown to increase sialic acid levels in the lumen and promoted the expansion of pathogenic bacteria such as C. difficile and Salmonella enterica serovar Typhimurium (42). Interestingly, sialic acid and other mucin sugar cleavage and transport expression were increased in BF+T mice along with an increased abundance of *Clostridium* XI and *Salmonella*. The SusC and SusD outer membrane proteins, involved in oligosaccharide binding and transport (43), were also increased in the BF+T community (from *Bacteroides* and *Parabacteroides* spp.). The upregulation of stress responses, mucin, and other nutrient acquisition genes indicate environmental conditions that could in turn promote virulence expression. Nutrient- and iron-responsive global transcriptional factors such as cyclic AMP receptor protein and ferric uptake regulator (Fur), which were increased in BF+T mice, have also been shown to regulate bacterial virulence expression (44).

Shotgun metagenomic sequencing of patient stools has revealed that host glycan utilization and virulence factor genes are associated with the CRC microbiome (16) and that genes in these categories were also overexpressed in the BF+T microbial community. Host inflammation, bacterial iron (Fur), and stress response (Hsp90 chaperone) genes have all been implicated in colibactin regulation, and all of these genes were increased in BF+T mice (45–47). Additionally, iron acquisition genes have previously been associated with E. coli mucus colonization (48), and mucins have the capacity to induce E. coli virulence gene expression (41). Mucin and its components may also serve as a cue for virulence regulation of other BF+T community members, as they have also been linked to virulence regulation in S. enterica and Campylobacter jejuni (41). Although the expression of multiple B. fragilis genes were increased in the BF+T community, B. fragilis toxin (*bft*) was not detected. One possible explanation is that expression of the RprXY two-component system, recently implicated in *bft* suppression (49), was significantly increased in BF+T mice. However, even intermittent enterotoxigenic B. fragilis colonization as short as 2 weeks appears to be sufficient to induce tumor formation (50), so it is possible that *bft* expression occurred at an earlier time point in the *Apc^Min^*^Δ^*^850/+^*;*Il10^−/−^* model. Taken together, the metatranscriptomic data suggest that the BF+T community expresses more pathogen-related virulence factors and metabolism genes that provide competitive advantages over commensals but may have detrimental side effects to the host.

Members of the *Erysipelotrichaceae* family, part of the core transmissible bacteria in BF+T mice, were also increased in the microbiota of Western or high-fat diet-fed mice (Clostridium innocuum, Eubacterium dolichum, and Clostridium ramosum) and were associated with increased fat deposition (51, 52). Diets high in fat and obesity are established risk factors for CRC (53). Conceivably, the activation of the PPAR signaling pathway within BF+T mice could be a response to colonization with these obesity-associated taxa. PPARs are nuclear hormone receptors that regulate key aspects of lipid and carbohydrate metabolism, including fatty acid synthesis, uptake, and storage (54), and have both suppressive and promotional effects in CRC (55).

Branched-chain amino acid (BCAA) biosynthesis was increased in the BF+T community, and serum BCAA have also been associated with metabolic disorders and correlated with intestinal microbiota members such as Bacteroides vulgatus (56). BCAAs, which include valine, leucine, and isoleucine, may also contribute to fatty acid synthesis, which is also a feature of cancer metabolism (57). Valine and/or leucine secretion were previously associated with E. coli (58) and polymicrobial environmental biofilms (59). Polyamines were previously shown through metabolomics to be increased in CRC patients, with a rare polyamine, N1, N12-diacetylspermine detected in biofilm-positive CRCs (60), and we found that multiple microbial polyamine-related genes were increased in BF+T mice (61). Polyamines increase in proliferating cells and can promote tumor growth and invasion (62) and are also important to bacterial biofilm formation (61). Several transporter genes (*Slc22a3*, *Abcb1a*, and *Abcb1b*) that have been previously implicated in polyamine uptake (63) were upregulated in BF+T mice. Thus, biofilm-associated communities and their associated metabolism pathways have the potential to modulate host metabolism, which may promote cancer.

In addition to bacterial components and metabolites, miRNAs represent another mode of host-microbe interplay during cancer. Profiling the fecal miRNAs of *Apc^Min^*^Δ^*^850/+^*;*Il10^−/−^* mice under different microbial conditions allowed us to identify specific miRNAs that were associated with biofilm/CRC status. A few of the CRC-associated miRNAs we identified have conserved sequences with human miRNAs (hsa-miR-21-5p, hsa-miR-142-5p, and hsa-miR-146a-5p) that are increased in CRC patients (17, 22, 64, 65). Mmu-miR-21a-5p was significantly increased in the BF+T mice, and F. nucleatum has previously been shown to increase miR-21 (22), suggesting that miR-21 may be targeted by multiple CRC-associated bacterial genera.

Although the depleted miRNAs in BF+T mice, miR-690 and miR-709, are not found in humans, they do share several CRC-related gene targets (such as *Ctnnb1*, *Il6ra*, *Stat3*, *Src*, and *Zeb1*) with other miRNAs depleted in CRC (17). Though none of these genes were significantly DE according to biofilm status in the mouse colon tissue RNA-seq data set, it is possible the luminal miRNAs might target other regions of the colon or specific intestinal epithelial cell types. Additionally, even though miRNAs have been shown to primarily control gene expression through mRNA degradation, translational repression is also possible (66). Alternatively, another mechanism of miRNA regulation could relate to targeting the RNA-induced silencing complex genes like *Argo1*, *Argo2*, *Argo3*, *Argo4*, *Cnot6*, and *Dcp2* (66), which are predicted targets of multiple significant DE miRNAs, the majority of which are increased in BF+T mice.

miRNAs also have the capacity to target bacterial genes and impact microbial composition (18), and our computational predictions indicate that newly discovered miRNAs (those with higher numbers in their names) preferentially target bacterial genes. These miRNAs include miR-2137, miR-5126, miR-6239, miR-6240, and miR-6538, which were also increased in DSS-treated mice (67), where microbiota composition also contributes to disease susceptibility (68). Many of these miRNAs were predicted to have redundant bacterial targets (including genes regulating motility, secretion, outer membrane proteins, stress response, iron acquisition, and carbohydrate utilization/transport) that overlap with genes that were increased in the BF+T microbial community. miR-6239 and -6240 were decreased in BF+T mice, but miR-2137, -5126, and -6538 were increased in mice, regardless of biofilm status.

### Conclusions.

**FIG 6:**
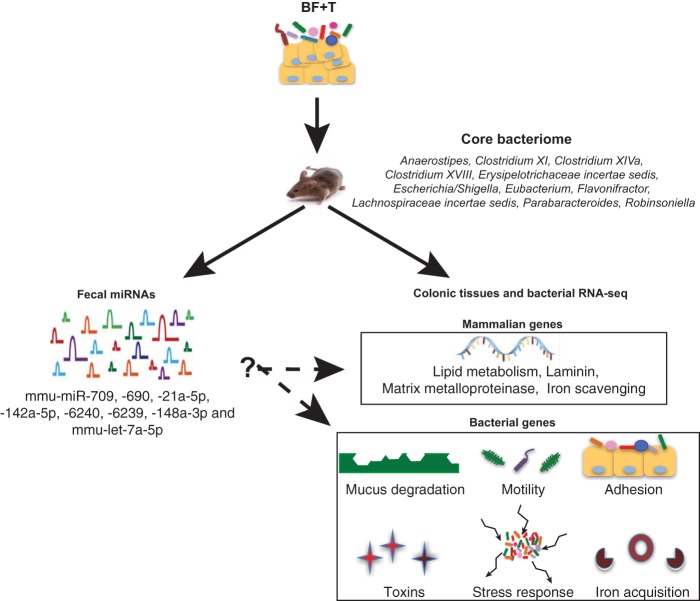
Schematic depiction of the major findings. The core, transmissible bacteria found in biofilm-positive tumor (BF+T) associated and reassociated mice are listed under core bacteriome. Some of the bacterial and mouse genes that were differentially expressed in the BF+T associated mice compared to biofilm-negative (BF-bx) associated mice are listed. Fecal miRNAs were also differentially expressed according to biofilm status, correlated with the relative abundances of some bacterial taxa, and were predicted to target mouse and bacterial genes.

The mechanisms by which miRNAs enter and regulate bacterial gene expression and how bacterial organization affects bacterial community gene expression warrant further investigation. A recent publication showed that plant-derived exosome-like nanoparticles contain miRNAs that altered intestinal microbial composition and gene function (69). Whether intestinal epithelial cell-derived exosomes carrying miRNAs could similarly target intestinal microbiota is under investigation. Our findings suggest a complex interplay between BF+T-associated bacteria, their gene expression, the host transcriptome, and miRNAs that may contribute to CRC pathogenesis (Fig. 6). Deciphering this complex interplay will likely identify new regulatory pathways and molecules with potential therapeutic implications.

## MATERIALS AND METHODS

### Animals.

Germfree (GF) 129/SvEv *Apc^Min^*^Δ^*^850/+^*;*Il10^−/−^* mice were transferred to separate gnotobiotic experimental isolators based on inoculum type for the duration of the association.

### Initial associations with human tissue-associated microbes.

GF 129/SvEv *Apc^Min^*^Δ^*^850/+^*;*Il10^−/−^* mice were inoculated with pooled tissue-derived microbes from biofilm-negative tissues collected from healthy patients via colonoscopy biopsy (BF-bx) or biofilm-positive tumor tissues collected from CRC patients (BF+T) via surgical resection. Patient tissues were collected and screened for biofilm status via fluorescence *in situ* hybridization (FISH) (biofilm-positive criteria, polymicrobial, within the mucus layer, spanning 200 μm, and >10^9^ bacteria/ml) as described previously (12). Tissues were analyzed with the universal bacterial probe (EUB338), and a nonsense probe (non338) was used as a negative control (12, 13). Additional FISH analysis was conducted on biofilm-positive tumor tissues with probes to detect *Bacteroidetes*, *Lachnospiraceae*, *Fusobacteria*, and *Proteobacteria* (12, 13). The probe sequences are listed in Table S4 in reference 12. Each inoculum was prepared anaerobically by homogenizing tissue (tissue pooled from five different patients) in phosphate-buffered saline (PBS), and FISH images for these tissues can be found in Fig. S1 in reference 13. Each mouse received 100 to 200 μl of inoculum, and associations were carried out for 12 to 20 weeks (Fig. 1AI). Tissues and/or stools from mice collected 12 weeks after association were used for transcriptome sequencing (RNA-seq), microRNA sequencing (miRNA-seq), and 16S rRNA gene sequencing analyses (Fig. 1AII).

### Mouse reassociation inoculums.

Mouse reassociation inoculums (Fig. 1AIII) were made from colon tissues from 12-week BF-bx-associated (cohort 2) and 16- to 20-week BF+T-associated (cohort 3) *Apc^Min^*^Δ^*^850/+^*;*Il10^−/−^* mice (13). After the colon was flushed 1× with PBS, tissue snips were taken from both the distal colon (DC) and proximal colon (PC) and stored at –80°C until time of inoculum preparation. Each inoculum was prepared from colon tissue snips pooled from four mice. All inoculums were prepared anaerobically by mincing and homogenizing tissue snips in prereduced PBS. The BF-bx reassociation inoculum was prepared from inflamed (average inflammation score of 2.5/4) distal colon tissues (BF-bx DC). The two BF+T reassociation inoculums were prepared from mostly normal (average PC inflammation score of 0.9/4) proximal colon tissues (BF+T PC) or distal colon tissues (BF+T DC) from the same four mice with colitis and tumors (average DC inflammation score of 3.6/4; average number of tumors = 5.5, range = 3 to 10 tumors).

### Reassociation with mouse tissue-associated microbes.

Six- to 13-week GF 129/SvEv *Apc^Min^*^Δ^*^850/+^*;*Il10^−/−^* (males and females) were transferred to gnotobiotic isolators (separate isolator for each experimental group) and gavaged with 100 to 200 μl of inoculum (Fig. 1AIII). Mice were euthanized after 12 weeks, and the colon was flushed once with PBS and then cut and splayed longitudinally for macroscopic tumor counts. About 2 × 0.5 cm tissue snips from the proximal and distal colon were collected by flash freezing in liquid nitrogen and stored at –80°C until analysis.

### Stool and distal colon tissue DNA extraction.

Stool and DC tissue DNA (Fig. 1AII and IV) was extracted via phenol-chloroform separation by lysing the cells with phenol-chloroform-isoamyl alcohol (25:24:1) and 0.1-mm zirconium glass beads on a bead beater (Precellys), followed by phase separation with chloroform-isoamyl alcohol (24:1), DNA precipitation with ethanol, and subsequent purification with the DNeasy Blood & Tissue kit (Qiagen catalog no. 69506).

### 16S rRNA sequencing.

The 16S rRNA V1-V3 hypervariable region was amplified using barcoded primer pairs 27F (5′-AGAGTTTGATCCTGGCTCAG-3′) and 534R (5′-ATTACCGCGGCTGCTGG-3′) with universal Illumina paired-end adapter sequences (see Tables S14 at https://figshare.com/s/ffd8c3066929eb0c69fc and S15 at https://figshare.com/s/5e95b9a855710812950b). PCR products were purified, quantified, and pooled as described previously (7) and sequenced with an Illumina MiSeq (as two separate runs). The first 16S rRNA run (Run01, Fig. 1AII and Table S16 at https://figshare.com/s/97c3b0a48482b6fb0ecf) included stool and DC tissue samples from BF-bx cohort 1, BF+T cohorts 1 to 3, and two different biofilm-positive groups (13). Differentially abundant taxa were identified by comparing stool (1- and 12-week time points) and DC tissue (12-week time point) communities collected from BF-bx #1, BF+T#1-2, and two additional biofilm-positive groups reported in Tomkovich et al. (13). The second 16S rRNA run (Run02, Fig. 1AII and IV and Table S16 at https://figshare.com/s/97c3b0a48482b6fb0ecf) included stool and DC tissue samples from BF-bx cohort 2 and the three reassociation groups (BF-bx DC, BF+T PC, and BF+T DC). Comparisons between initial associations and reassociations were assessed by comparing the microbiota from mice whose tissues were used for the inoculums (BF-bx #2 and BF+T#3) to the reassociation microbiotas (BF-bx DC, BF+T PC, and BF+T DC).

### 16S rRNA sequencing analysis.

Reads were preprocessed using Quantitative Insights into Microbial Ecology (QIIME) (70) version 1.9.1 including trimming and filtering at Q20. The final set of reads was fed to the RDP (Ribosomal Database Project) classifier (71) version 2.12 with the confidence set at 80%. Reads were grouped by genera, and samples with less than 1,000 total reads and genera with less than 5 reads were removed. The resulting counts were normalized and log_10_ transformed (72) using the following formula:

$$\log_{10}\left(\frac{RC}{n}\times \frac{\Sigma x}{N}+1\right)$$

where *RC* is the read count for a particular taxon in a particular sample, *n* is the total number of reads in that sample, the sum of *x* is the total number of reads in all samples, and *N* is the total number of samples. The principal-coordinate analysis (PCoA) was generated from the Bray-Curtis distance of the normalized and log_10_-transformed counts using the phyloseq (73) R package (74).

Genera significant for biofilm group (BF-bx, BF+T, BF-bx DC, BF+T PC, and BF+T DC) were detected using the lme function in the R nlme package, with the REML method (75) to fit a mixed linear model of the form:

genus∼variable+1|cage+ε

where genus indicates the log_10_ normalized abundance of a particular genera, variable indicates either the biofilm group or PCoA axis, and 1|cage indicates that we used the cage as a random effect. We then ran an analysis of variance (ANOVA) on the above model to generate *P* values for biofilm group or PCoA axis. We checked for possible cage effect by comparing the above model and a model with the cage removed (genus ∼ variable + ɛ) using ANOVA. The *P* values were adjusted for multiple hypothesis testing in R using the p.adjust function employing the method of Benjamini and Hochberg (76). The heatmaps were generated using the R function ggplot2 (77).

We performed two additional analyses on the 16S rRNA data, the first utilizing QIIME (70) v. 1.9.1 closed-reference at 97% similarity level using the Greengenes reference data set release 13_8 and the second employing Deblur (78) workflow v. 1.0.3 with the default parameters (using Deblur’s default positive and negative reference filtering) and trim length set to 100 bases. Both pipelines showed no significant separation between the BF-bx and BF-bx DC samples (see Fig. S4C and D in the supplemental material).

### RNA extraction, rRNA depletion, and RNA sequencing.

Total RNA was extracted from frozen proximal colon tissue snips (Fig. 1AII) using the mirVana miRNA isolation kit, with phenol (ThermoFisher Scientific catalog no. AM1560) according to the manufacturer’s instructions, with the addition of an ∼1:1 mix of 1-mm acid-washed glass beads and 0.1-mm zirconia beads and a Precellys24 (Bertin Instruments catalog no. EQ03119-200-RD000.0) bead beater for tissue disruption and lysis. Extracted RNA was treated with the Turbo DNA-free kit (ThermoFisher Scientific catalog no. AM1907) to remove DNA. Quality control, rRNA depletion, and cDNA library preparation were performed by the University of Florida’s Interdisciplinary Center for Biotechnology Research (ICBR) Gene Expression and Genotyping core using the Agilent 2100 bioanalyzer (Agilent Genomics catalog no. G2939BA), Ribo-Zero Gold rRNA removal kit (Epidemiology) (Illumina catalog no. MRZE724) and ScriptSeq v2 RNASeq library preparation kit (Illumina catalog no. SSV21124) starting with 1 μg total RNA. Samples were sequenced by the University of Florida ICBR NextGen DNA Sequencing core on the Illumina HiSeq 3000 (2 × 100 run), multiplexing each sample into three lanes to avoid lane effect.

### Mouse RNA-seq analysis.

Reads were quality filtered at Q20 and trimmed to remove remaining adapters using Trimmomatic (79) version 0.36. The resulting reads were aligned to Illumina iGenome Mus musculus Ensembl GRCm38 reference genome using Tophat (80) version 2.1.1 utilizing Bowtie2 (81) version 2.3.0 following the approach of Gilad and Mizrahi-Man (82). The resulting alignments (averaging 34,079,158 concordant read pairs per sample) were processed using Cufflinks (83) version 2.2.1 along with Illumina iGenome Mus musculus Ensembl GRCm38 gene transfer format file, after masking rRNA features (82). We used cuffquant to perform transcript quantification and exported the raw counts (nonnormalized counts) to text files. The raw counts were then imported to edgeR (84) version 3.16.5 for detecting differentially expressed (DE) genes. A gene was considered for the differential expression test if it was present in at least 50% of the samples. We considered a gene DE if its edgeR false-discovery rate (FDR)-adjusted *P* value (*P*_FDR_) was <0.05. Parallel analysis using featureCounts from the subread package version 1.5.3 for transcript quantification showed similar results (data not shown) (85). Pathway analysis was conducted through GAGE (86) version 2.24 using Mus musculus (mmu) Kyoto Encyclopedia of Genes and Genomes (KEGG) (87) pathways, and genes were mapped to KEGG pathways using Pathview (88). We considered a pathway significant if its GAGE false-discovery rate (*q* value) was less than 0.05. We tested the effect of sequencing lane on the clustering of the samples (Fig. 2A) and found it to be insignificant (*P* value > 0.05) (data not shown).

### Metatranscriptome analysis.

Quality-filtered and trimmed reads from above were aligned to iGenome Mus musculus Ensembl GRCm38 reference genome using BWA (89) version 0.7.16a, and reads with alignments were excluded from further analysis. The remaining reads were then filtered from rRNA and tRNA sequences by aligning (using BWA) to a collection of NCBI rRNA and tRNA sequences and SILVA database sequences, resulting in an average of 1,208,429 reads per sample, which were then submitted for *de novo* assembly using Trinity (90) version 2.4.0. The resulting assembly was annotated using Trinotate (91) version 3.0.1 (http://trinotate.github.io) with the following databases: uniprot_sprot (92), Pfam (93), and Virulence Factor Database (VFDB) (94). The resulting annotations were examined, and sequences annotated as nonbacterial were removed. Transcript abundance was determined using RNA-seq by expectation maximization (RSEM) (95) through Trinity’s align_and_estimate_abundance.pl script, and the counts were imported to edgeR version 3.16.5 for differential expression analysis. A gene was considered for the differential expression test if it was present in at least 50% of the samples. We considered a transcript DE if its edgeR FDR-adjusted *P* value was <0.05. To account for normalization artifacts, we also examined the ratios of DE genes between the BF+T and BF-bx groups generated from a rarefied data set that was based on 140,000 reads per sample (Table 2). The similar ratios of DE genes from the complete and rarefied data sets suggest that our findings are not an artifact of normalization. We conducted a second analysis (reference-based analysis) by aligning the reads submitted for *de novo* assembly to the human gut microbiome integrated gene catalog (IGC) (96) using Bowtie2 (81) (v.2.3.4.2) followed by quantification using featureCounts (85) from the subread package (v.1.5.3) and obtained similar results (Fig. S2A).

**TABLE 2:** Ratios of DE genes in BF+T and BF-bx groups

Data set	% of genes		
	DE genes (FDR < 0.05) (% of input transcripts)	Upregulated genes in BF+T group (% of DE transcripts)	Downregulated genes in BF+T group (% of DE transcripts)
Complete	36	93	7
Rarefied^*a*^	40	88	12

a The rarefied data set was based on 140,000 reads per sample.

Pathway analysis was conducted through GAGE version 2.24 using Kyoto Encyclopedia of Genes and Genomes (KEGG) reference pathways on the assembled transcript and The Human Microbiome Project (HMP) Unified Metabolic Analysis Network (HUMAnN) (97) on the unassembled reads. Genes were mapped to KEGG pathways using Pathview (88). We considered a pathway significant if its *q* value was <0.05. Weighted gene coexpression network analysis (WGCNA) version 1.68 (23) was utilized to detect modules in each biofilm status samples using the blockwiseConsensusModules function which performs the network construction and consensus module detection. The hub gene in each detected module was identified using the WGCNA function chooseTopHubInEachModule. The sequencing lane had no effect on the clustering of the samples in Fig. 1A (*P* value > 0.05, data not shown).

### miRNA extraction and sequencing.

Small RNAs were extracted from snap-frozen stool samples (Fig. 1AII) using the mirVana miRNA isolation kit. Because of the low amount of small RNA, GF stools were pooled from 13 *Apc^Min^*^Δ^*^850/+^*;*Il10^−/−^* mice (20- to 44-week age range) total, or stools from 2 to 5 mice per sample (*n* = 4). BF-bx and BF+T small RNAs were extracted from the stools of 12-week-associated BF-bx (*n* = 7) and BF+T (*n* = 10) *Apc^Min^*^Δ^*^850/+^*;*Il10^−/−^* mice. cDNA libraries were synthesized with the NEBNext Multiplex Small RNA Library Prep Set for Illumina kit (New England Biolabs catalog no. E7300) and small RNAs for each library (21- to 30-nucleotide size range) were gel purified. For the GF, BF-bx, and BF+T comparisons, a pool of 21 libraries (equivalent molar concentrations; 4 GF, 7 BF-bx, and 10 BF+T) were multiplexed and sequenced using the Illumina Miseq.

### miRNA analysis.

CAP-miRSeq (98) was used to process the miRNA sequences. We used the databases and reference sequences that ship with CAP-miRSeq for all the analyses. Briefly, sequences were filtered and trimmed using cutadapt (99). Quantification of miRNA was done using miRDeep2 (100), and DE miRNAs were detected using edgeR version 3.16.5. We considered a miRNA DE if its edgeR FDR-adjusted *P* value was <0.05. Principal-component analysis (PCA) was created using R’s prcomp function from the normalized and log_10_-transformed miRNA counts according to the equation above.

Correlations with microbiota taxon abundance were done using R lm function, *P* values were determined using R’s ANOVA function, and FDR correction was done using R’s p.adjust function employing the method of Benjamini and Hochberg (76) and only those with a FDR-adjusted *P* value of <0.05 were considered.

Mouse miRNA targets were predicted using miRDB (101), and bacterial target prediction for mice miRNA was done using PITA (102) on the assembled bacterial transcripts from the RNA-seq data described above. We considered a bacterial transcript a potential target for a particular mouse miRNA if its ΔΔ*G* score was less than or equal to −15 kcal/mol. The bacterial and mouse gene targets of miRNAs significantly different between GF, BF-bx, and BF+T groups are listed in Tables S12 at https://figshare.com/s/33fb65da16763a5e3347 and S13 at https://figshare.com/s/be3f38607b2e9875e7f6.

For miRNA expression correlation with tumor numbers, two BF-bx samples were excluded because they were fixed without splaying the colon so tumor counts could not be generated. Correlation was done using Spearman’s rank correlation through R’s cor.test function.

### Statistical analyses.

For all sequencing analyses, a taxon or miRNA was considered only if it was present in at least 30% of the comparison samples, and statistics are described in above 16S rRNA sequencing, mouse RNA-seq, metatranscriptome and miRNA analysis sections. *P* values of <0.05 were considered statistically significant.

### Ethics.

All animal experiments were approved by the University of Florida Institutional Animal Care and Use Committee (protocol 201308038). All patient tissues were collected as previously described (11, 12, 103).

### Data availability.

The data supporting the results of this article have been deposited in the National Center for Biotechnology Information Gene Expression Omnibus (NCBI GEO) under accession number GSE108165 for the RNA-seq and miRNA data sets and the National Center for Biotechnology Information Sequence Read Archive (NCBI SRA) under BioProject identifier (ID) PRJNA422588 for 16S rRNA Run02 sequences. Run01 sequences are in the article by Tomkovich et al. (13).
