# Supplementary material for: Human Colon Mucosal Biofilms and Murine Host Communicate via Altered mRNA and microRNA Expression during Cancer
Source: mSystems. 2020 Jan 14;5(1):e00451-19. doi: 10.1128/mSystems.00451-19 (PMC6967385; doi:10.1128/mSystems.00451-19)
Supplement: FIG S1 [file mSystems.00451-19-sf001.pdf]

Figure S1

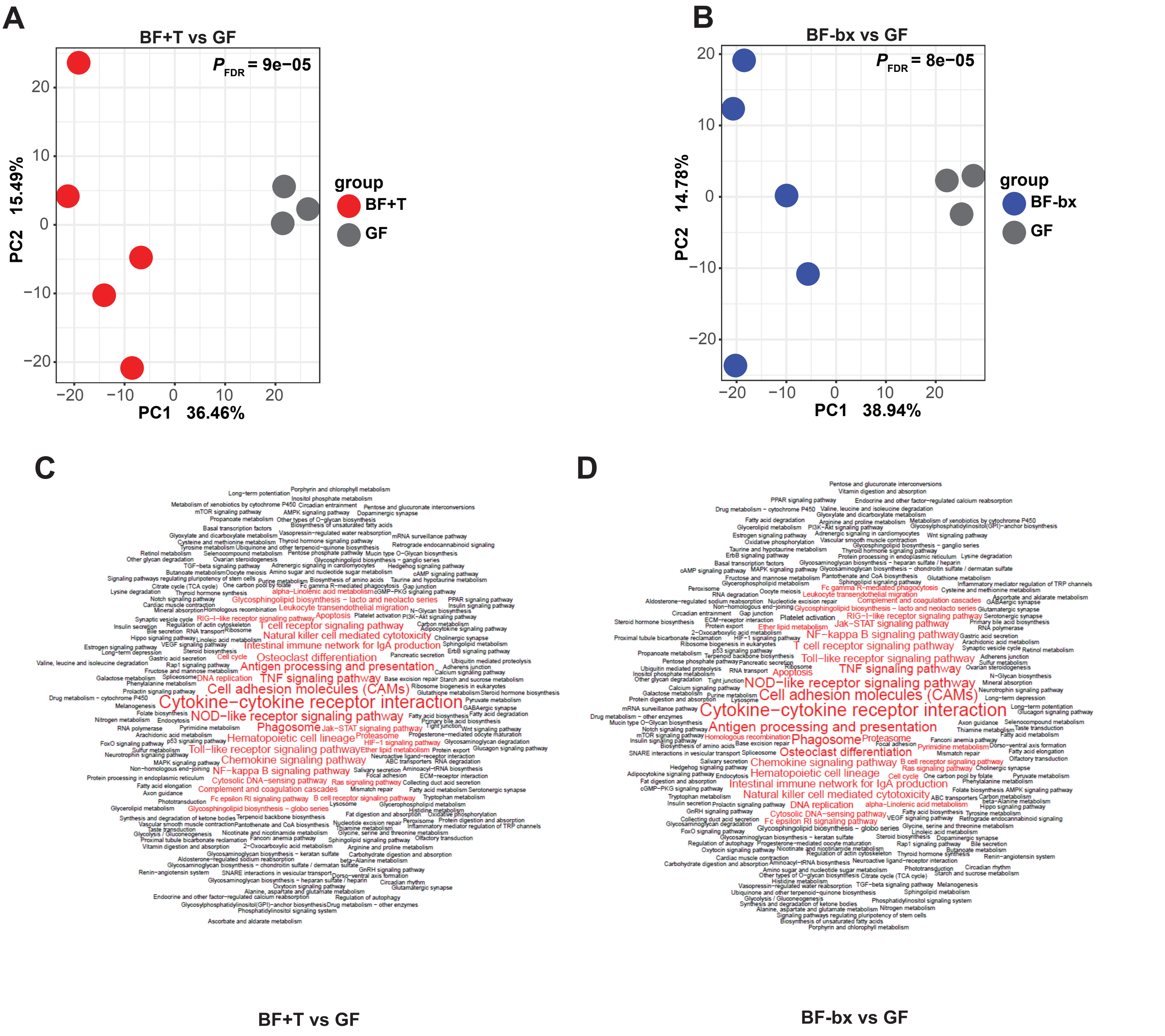

A
BF+T vs GF
P_{FDR} = 9e−05
PC2 15.49%
PC1 36.46%
group
BF+T
GF
B
BF-bx vs GF
P_{FDR} = 8e−05
PC2 14.78%
PC1 38.94%
group
BF-bx
GF
C
Cytokine−cytokine receptor interaction
NOD−like receptor signaling pathway
Cell adhesion molecules (CAMs)
TNF signaling pathway
Antigen processing and presentation
Osteoclast differentiation
Intestinal immune network for IgA production
Natural killer cell mediated cytotoxicity
T cell receptor signaling pathway
Phagosome
Hematopoietic cell lineage
Toll−like receptor signaling pathway
Chemokine signaling pathway
NF−kappa B signaling pathway
BF+T vs GF
D
Cytokine−cytokine receptor interaction
Cell adhesion molecules (CAMs)
NOD−like receptor signaling pathway
Antigen processing and presentation
Phagosome
Osteoclast differentiation
Chemokine signaling pathway
Hematopoietic cell lineage
Intestinal immune network for IgA production
Natural killer cell mediated cytotoxicity
TNF signaling pathway
Toll−like receptor signaling pathway
T cell receptor signaling pathway
NF−kappa B signaling pathway
BF-bx vs GF
