# Supplementary figures and images for: Human Colon Mucosal Biofilms and Murine Host Communicate via Altered mRNA and microRNA Expression during Cancer
Source: mSystems. 2020 Jan 14;5(1):e00451-19. doi: 10.1128/mSystems.00451-19 (PMC6967385; doi:10.1128/mSystems.00451-19)

Figure S2

A

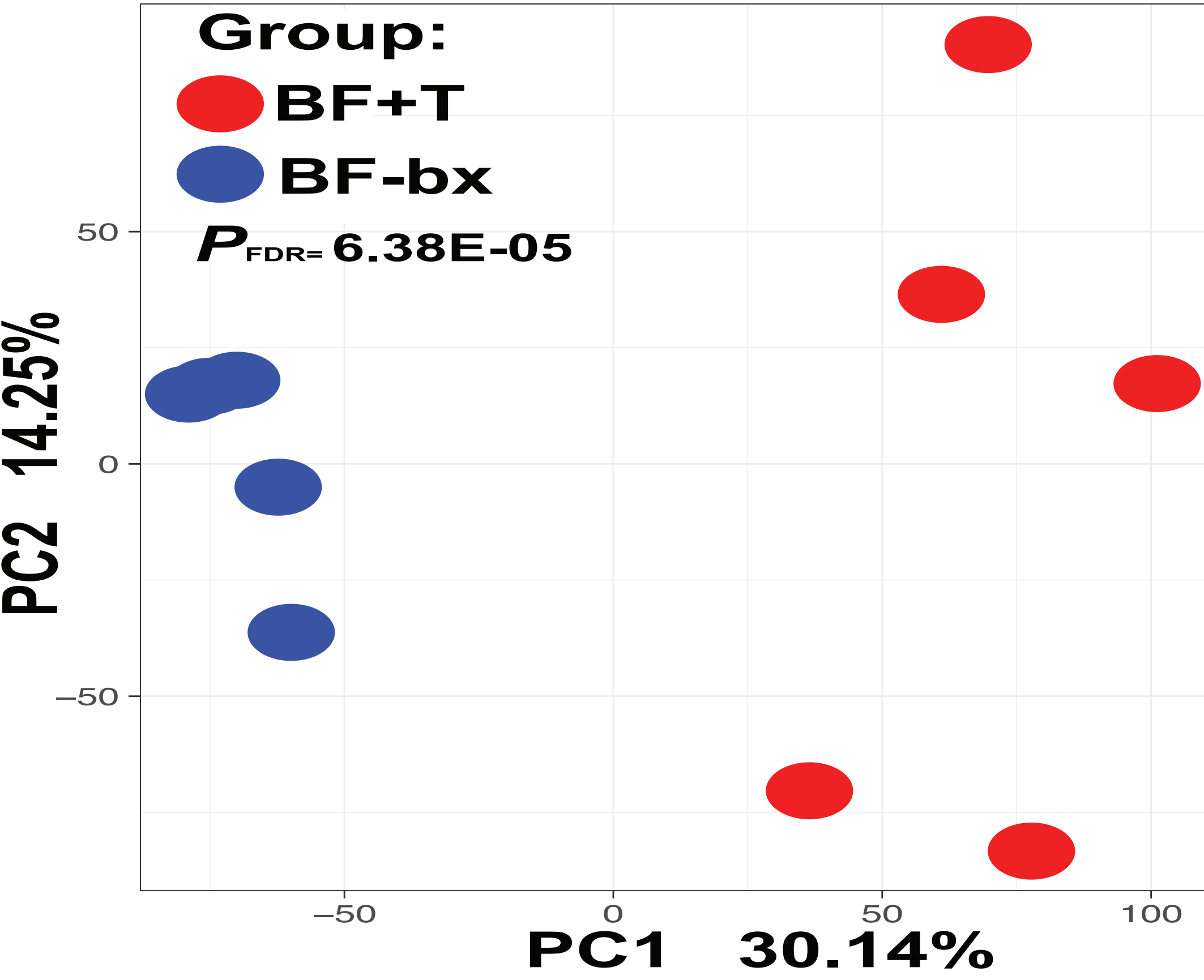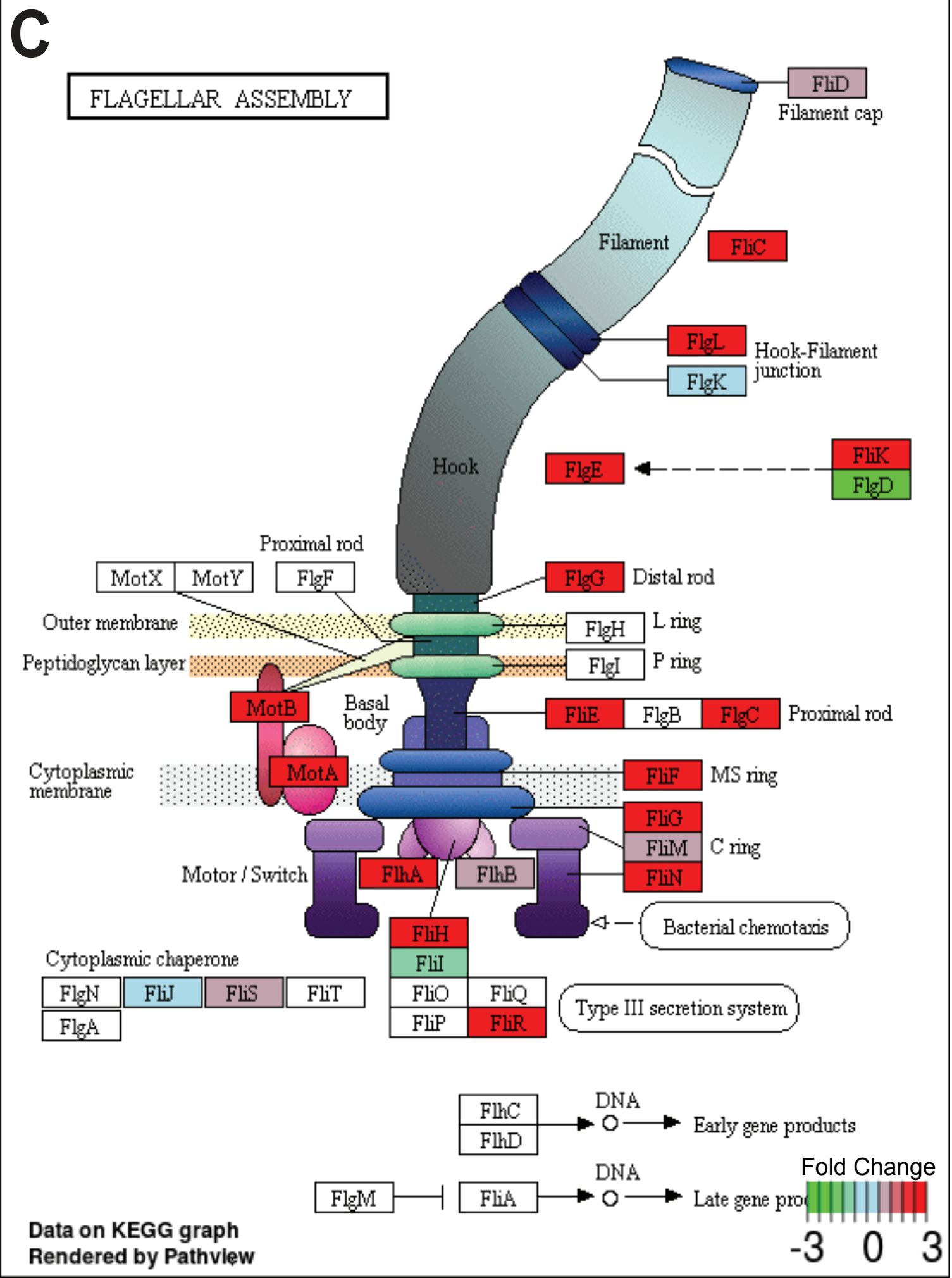

B

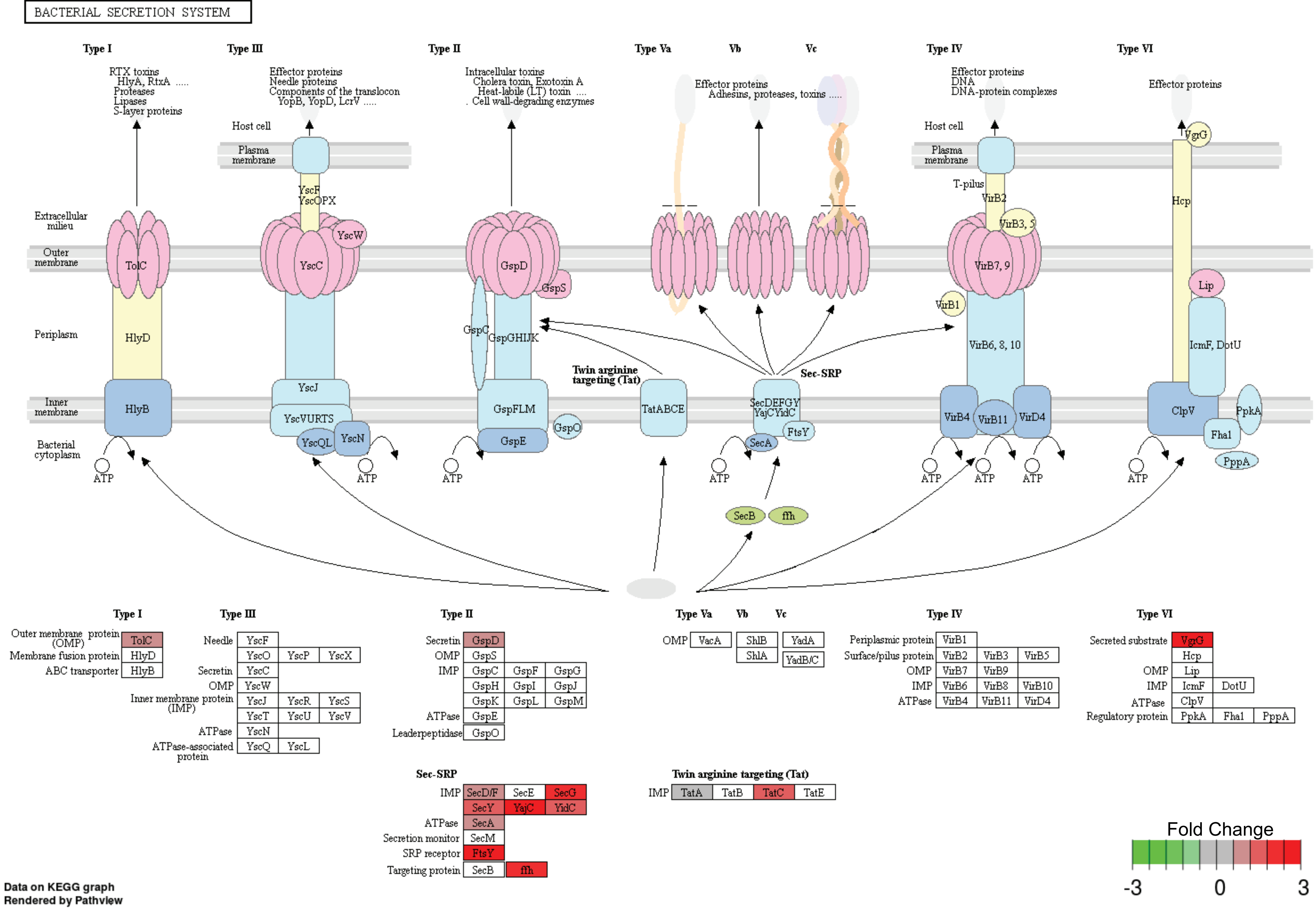

Supplement: FIG S2 [file mSystems.00451-19-sf002.pdf]

Figure S3

A

miRNA correlation with tumor number

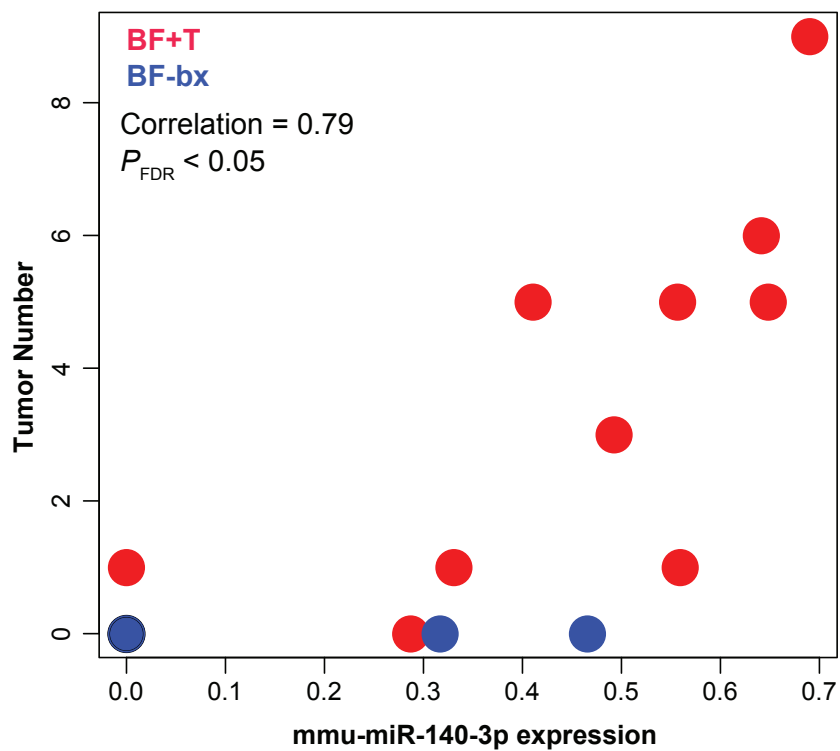100 randomly selected sets of miRNAs

B

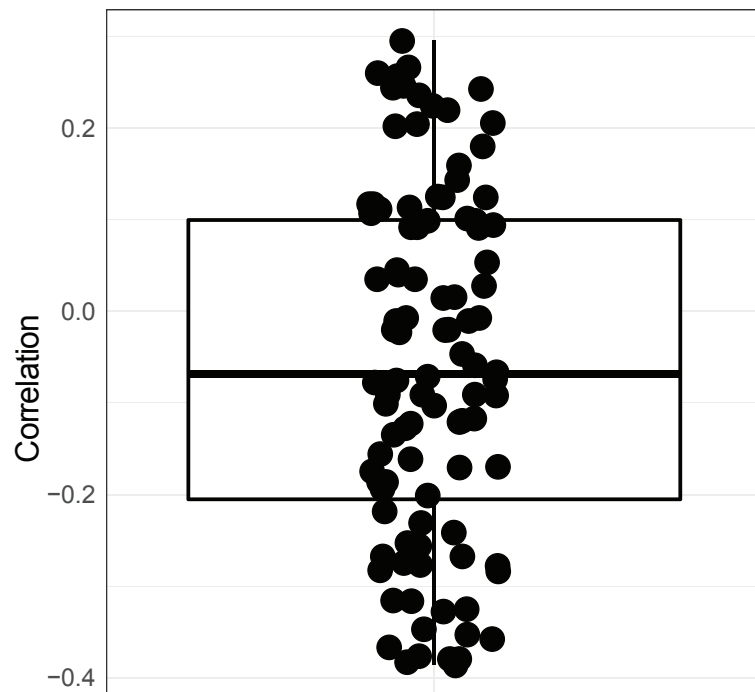

C

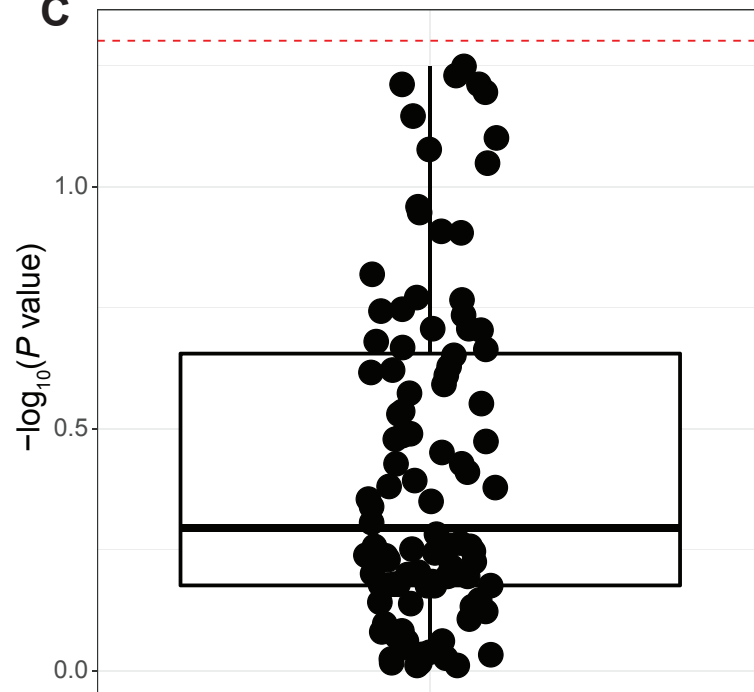

Supplement: FIG S3 [file mSystems.00451-19-sf003.pdf]

Figure S6

A

**BF+T reassociation comparison**

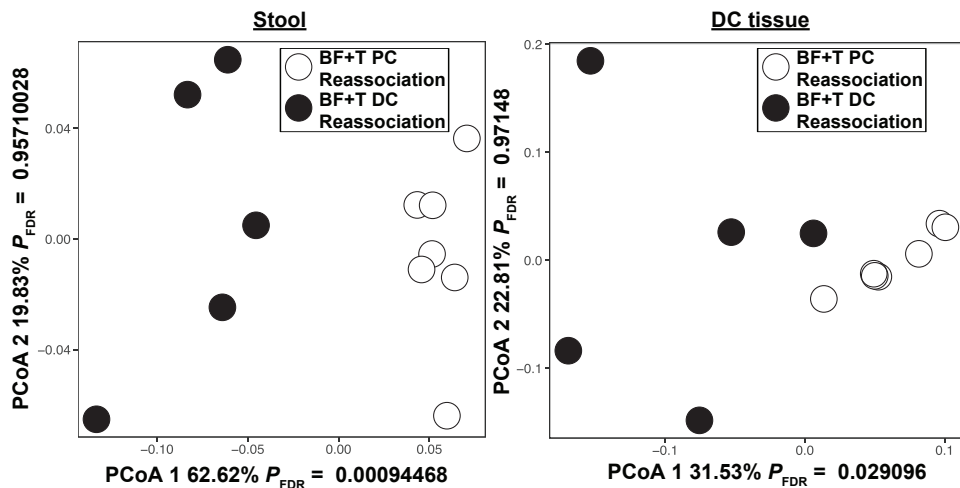

B

**Significantly different genera**

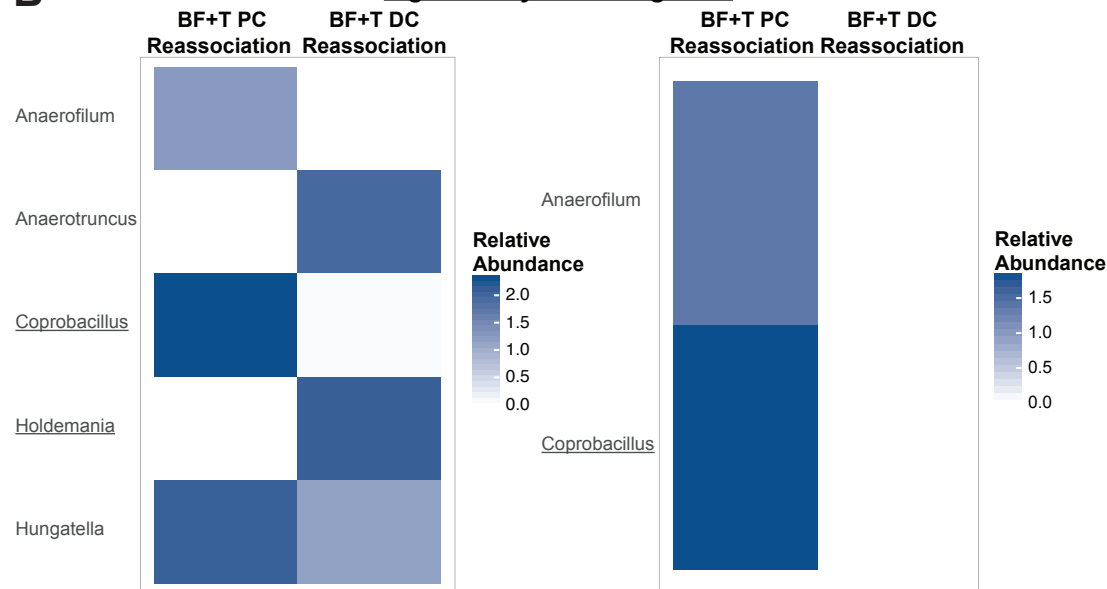

Supplement: FIG S6 [file mSystems.00451-19-sf006.pdf]
