# Supplementary material for: Human Colon Mucosal Biofilms and Murine Host Communicate via Altered mRNA and microRNA Expression during Cancer
Source: mSystems. 2020 Jan 14;5(1):e00451-19. doi: 10.1128/mSystems.00451-19 (PMC6967385; doi:10.1128/mSystems.00451-19)
Supplement: FIG S4 [file mSystems.00451-19-sf004.pdf]

Figure S4

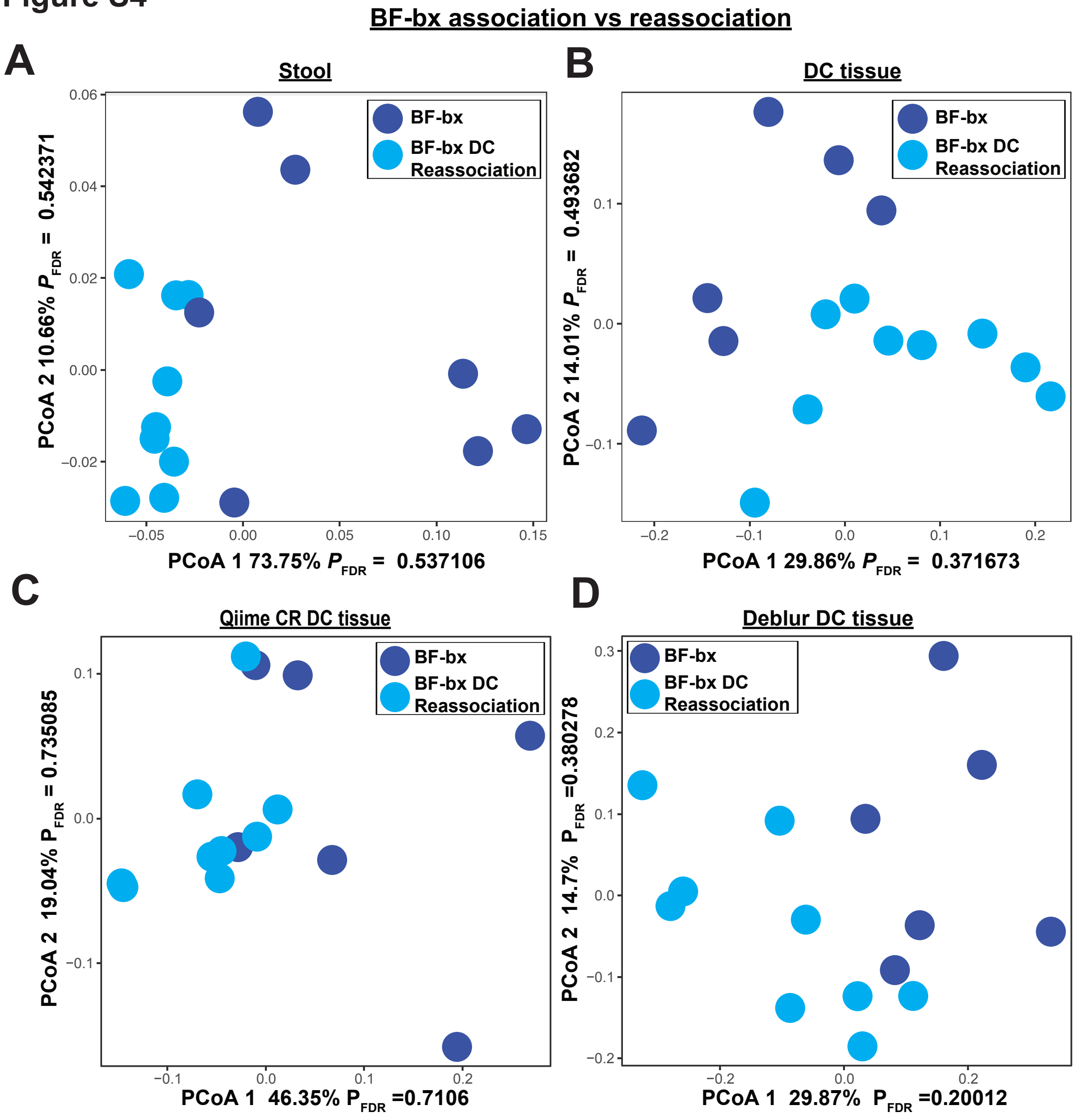
BF-bx association vs reassociation
A
Stool
BF-bx
BF-bx DC Reassociation
PCoA 2 10.66% P FDR = 0.542371
PCoA 1 73.75% P FDR = 0.537106
B
DC tissue
BF-bx
BF-bx DC Reassociation
PCoA 2 14.01% P FDR = 0.493682
PCoA 1 29.86% P FDR = 0.371673
C
Qiime CR DC tissue
BF-bx
BF-bx DC Reassociation
PCoA 2 19.04% P FDR = 0.735085
PCoA 1 46.35% P FDR =0.7106
D
Deblur DC tissue
BF-bx
BF-bx DC Reassociation
PCoA 2 14.7% P FDR =0.380278
PCoA 1 29.87% P FDR =0.20012
