# Supplementary material for: Human Colon Mucosal Biofilms and Murine Host Communicate via Altered mRNA and microRNA Expression during Cancer
Source: mSystems. 2020 Jan 14;5(1):e00451-19. doi: 10.1128/mSystems.00451-19 (PMC6967385; doi:10.1128/mSystems.00451-19)
Supplement: FIG S5 [file mSystems.00451-19-sf005.pdf]

Figure S5

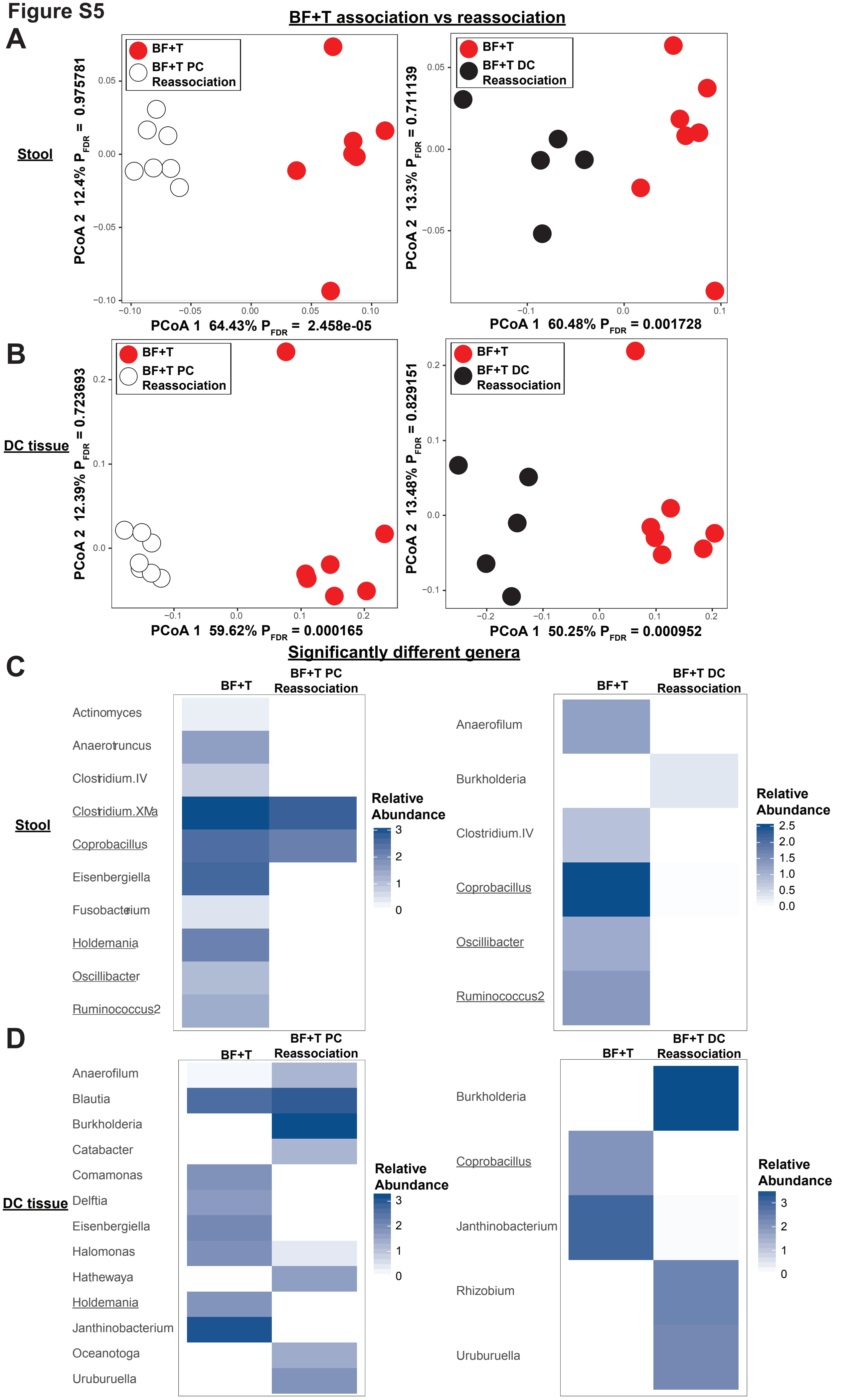

BF+T association vs reassociation
A
Stool
BF+T
BF+T PC Reassociation
BF+T DC Reassociation
PCoA 2 12.4% P FDR = 0.975781
PCoA 1 64.43% P FDR = 2.458e-05
PCoA 2 13.3% P FDR = 0.711139
PCoA 1 60.48% P FDR = 0.001728
B
DC tissue
PCoA 2 12.39% P FDR = 0.723693
PCoA 1 59.62% P FDR = 0.000165
PCoA 2 13.48% P FDR = 0.829151
PCoA 1 50.25% P FDR = 0.000952
Significantly different genera
C
Stool
Actinomyces
Anaerotruncus
Clostridium.IV
Clostridium.XIVa
Coprobacillus
Eisenbergiella
Fusobacterium
Holdemania
Oscillibacter
Ruminococcus2
Relative Abundance
Anaerofilum
Burkholderia
Clostridium.IV
Coprobacillus
Oscillibacter
Ruminococcus2
D
DC tissue
Anaerofilum
Blautia
Burkholderia
Catabacter
Comamonas
Delftia
Eisenbergiella
Halomonas
Hathewaya
Holdemania
Janthinobacterium
Oceanotoga
Uruburuella
Burkholderia
Coprobacillus
Janthinobacterium
Rhizobium
Uruburuella
